# Developing an Implicit Solvation Machine Learning Model for Molecular Simulations of Ionic Media

**DOI:** 10.1021/acs.jctc.3c00984

**Published:** 2023-12-20

**Authors:** Amaury Coste, Ema Slejko, Julija Zavadlav, Matej Praprotnik

**Affiliations:** † Laboratory for Molecular Modeling, National Institute of Chemistry, Ljubljana SI-1001, Slovenia; ‡ Professorship of Multiscale Modeling of Fluid Materials, TUM School of Engineering and Design, Technical University of Munich, Garching Near Munich DE-85748, Germany; § Department of Physics, Faculty of Mathematics and Physics, University of Ljubljana, Ljubljana SI-1000, Slovenia

## Abstract

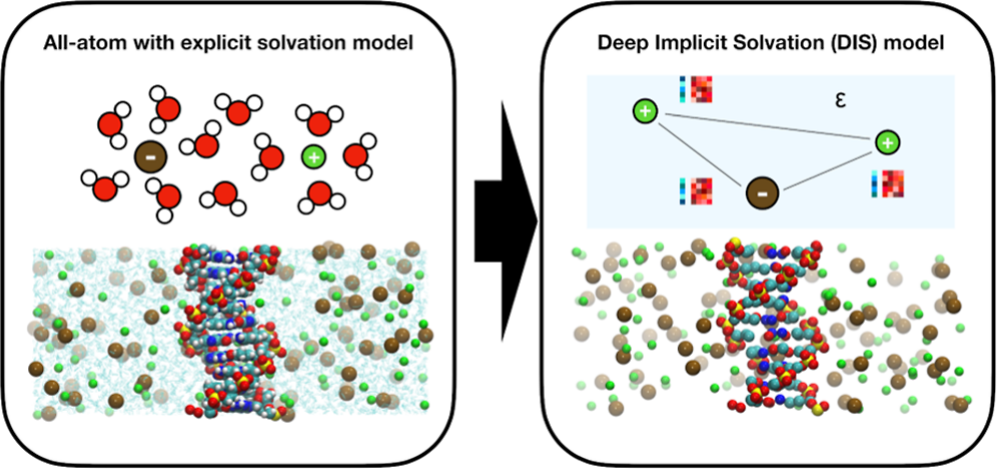

Molecular dynamics (MD) simulations of biophysical systems require accurate modeling of their native environment, i.e., aqueous ionic solution, as it critically impacts the structure and function of biomolecules. On the other hand, the models should be computationally efficient to enable simulations of large spatiotemporal scales. Here, we present the deep implicit solvation model for sodium chloride solutions that satisfies both requirements. Owing to the use of the neural network potential, the model can capture the many-body potential of mean force, while the implicit water treatment renders the model inexpensive. We demonstrate our approach first for pure ionic solutions with concentrations ranging from physiological to 2 M. We then extend the model to capture the effective ion interactions in the vicinity and far away from a DNA molecule. In both cases, the structural properties are in good agreement with all-atom MD, showcasing a general methodology for the efficient and accurate modeling of ionic media.

## 1 Introduction

Molecular dynamics (MD) is a powerful computational technique to understand and predict the behavior of biological systems, such as nucleic acids, proteins, lipid membranes, and many others.^1−15^ The all-atom MD explicitly models all atoms in the simulated system. However, since the computational cost scales with the number of atoms, the method is often inapplicable to biologically relevant time scales and system sizes. Approaches like enhanced sampling techniques^16−19^ can overcome this limitation to some extent.

Alternatively, to reduce the computational complexity, coarse-grained (CG) modeling^20−25^ can be employed. It involves reducing the simulated degrees of freedom either by merging groups of correlated atoms into effective interaction sites or by treating part of the system, typically solvent, implicitly. The latter case can drastically reduce the number of explicit particle–particle interactions and the resulting computational costs since the solvent can comprise more than 90% of the simulated system. The solvent, treated as a dielectric continuum, can be considered with methods such as the Poisson–Boltzmann (PB),^26,27^ COSMO/polarized continuum model,^28^ the Generalized Born model,^29−31^ or calculating the effective potential between solutes.^32^ However, the accuracy of these methods is often inadequate, e.g., some cannot maintain stable nucleic acid structures, or they introduce structural bias in proteins.^33,34^

Nevertheless, with multiscale simulations, one can employ CG models in the bulk region, while more accurate all-atom models can be used in the vicinity of biomolecules. Using the adaptive resolution simulation scheme,^35−38^ the solvent, i.e., water molecules and ions, can change the resolution on-the-fly from all-atom to CG^39^ or implicit hydration^40^ and vice versa. This approach was, for example, employed to efficiently simulate a DNA molecule^38,40^ and to study the mechanism governing the phase transitions of the high-density DNA arrays.^41,42^

Machine learning (ML) paves the way for a new possibility in the past decade.^43^ The pioneering work of Behler showcased that deep neural networks (NN) could be employed to learn a computationally cheaper surrogate model for the density functional theory potential energy surface of bulk silicon.^44^ Following the initial studies,^45^ other ML algorithms and NN architectures rapidly emerged.^46−55^ ML algorithms can be utilized to construct not only all-atom models but also CG models.^56,57^ Previous studies demonstrated a successful reproduction of structural^58−64^ and dynamical^65−67^ properties. However, most considered pure water solvent or implicitly treated the ions, even though they play a vital role in biological processes. For example, the ionic atmosphere has a crucial effect on the secondary and tertiary structure’s stability, the binding of charged drugs and proteins, and nucleic acid folding.^68−70^ On the other hand, previous ML potentials that captured the explicit interaction of ions also explicitly treated the solvent, i.e., they were fully all-atom models.^71−75^

Several approaches were developed to fit the potential of mean force, which corresponds to a high-dimensional energy function. These methods are the iterative Boltzmann inversion (IBI),^76,77^ the force matching,^78−80^ and the relative entropy.^81^ These methods have been widely used to determine the nonbonded interaction for aqueous salt solutions^82,83^ and soft matter.^77^ The resulting potentials are pairwise. However, for the force-matched potential, it has been shown that including 3-body nonbonded interaction has an impact on structural and thermodynamic properties.^84^ For the calculation of a CG protein force field, the importance of higher-order terms was demonstrated to reach accuracy close to the all-atom model.^85,86^ As stated before, ML potentials are designed to learn the many-body atomic interaction.^87,88^ They are, by design, more adequate to approximate the many-body terms of the potential of the mean force.

Another crucial point regarding the simulation of ions and highly charged molecules is the electrostatic interactions.^89^ For example, without ions in solution, the effective DNA–DNA interactions are purely repulsive.^90^ This implies an accurate description of long-range interactions. Most of the ML potentials learn the local geometry of atoms around a central one until a defined cutoff distance. The potentials are thus “short-ranged” and neglect long-range physical effects. For isotropic systems such as water solutions, for example, the ML potential without an explicit electrostatic term already gives excellent results.^91^ On the contrary, for battery materials, it was shown that a long-range electrostatic treatment is needed when the system is anisotropic.^92^ Anisotropic environments for solutes are also observed in biological systems and in other interfacial systems. To address this major drawback, different ML architectures explicitly describe the electrostatic energy such as charge equilibration,^93^ the long-distance equivariant framework LODE,^43,94^ and the fourth-generation potential.^53,95^ Another way is to use a delta-learning approach where the physics-based potential captures the long-range interactions.^96−98^

This work presents a deep implicit solvation (DIS) model for sodium chloride solutions, where water is coarse-grained out, whereas ions are modeled explicitly. The ML potential is based on an equivariant neural network (ENN) architecture due to its impressive data efficiency and the ability to generalize more accurately to out-of-distribution configurations.^51,52,54,55,99,100^ As stated by the Allegro’s developers “the strict locality of the Allegro model naturally facilitates separation of the energy into a short-range term and a physically motivated long-range term.” Thus, rather than directly fitting the potential of mean force, we define a prior potential composed of the Lennard-Jones and electrostatic interactions. The ML potential is trained to account for the difference between the all-atom data and the prior potential, an approach also known as delta learning.^101^ Our model can, therefore, account for the long-range electrostatic interactions, which are crucial for the accurate treatment of ions and highly charged molecules such as DNA. While some previous ML potentials included long-range electrostatics,^53,102,103^ these models were computationally much more expensive. First, we validate the sodium chloride aqueous solution model at different salt concentrations ranging from 0.15 to 2.0 mol L^–1^. After showing excellent performance for structural properties, we introduce a periodic DNA molecule into the system and demonstrate that our DIS model can accurately describe the effective ion interactions proximal and distal to the DNA biomolecule.

## 2 Methods

### 2.1 Database Generation

We performed classical all-atom simulations to obtain a database for the DIS model training and validation. These simulations were also used to compute the reference all-atom properties. We employ the TIP3P water model^104^ and AMBER 03 force field^105^ for the DNA molecule. The sequence of the periodic DNA molecule is CTCTCGAGAG. The Joung and Cheatham parameters^106^ are used for the ions with additional corrections to ion–phosphate interactions.^107^ The nonbonded interactions are calculated within cutoff distances of 0.9 and 1.2 nm, respectively, for the LJ and the electrostatic potential. The electrostatic interactions beyond the cutoff are corrected with the PPPM solver.^108^ Since the aim of this study was focused on obtaining an accurate many-body potential of mean force for the ions, the atoms of the DNA molecule were frozen. A flexible DNA pitch could also be considered. All simulations are equilibrated in the *NPT* ensemble for 2 ns, followed by equilibrations in the *NVT* ensemble of 10 and 15 ns for 1.0 and 0.5 mol L^–1^, respectively. During the production runs, carried out in the *NVT* ensemble, the forces applied on ions are saved every 1 ps. For pure salt solution systems, the number of configurations in the data set varies from 8 × 10^4^ for 2.0 mol L^–1^ salt concentration to 3 × 10^5^ for 0.15 mol L^–1^ salt concentration. For systems with the DNA molecule, the data set contains 1.8 × 10^5^ configurations. For all cases, we randomly split the data set into training (80%) and validation (20%) data sets.

### 2.2 DIS Model

**Figure 1:**
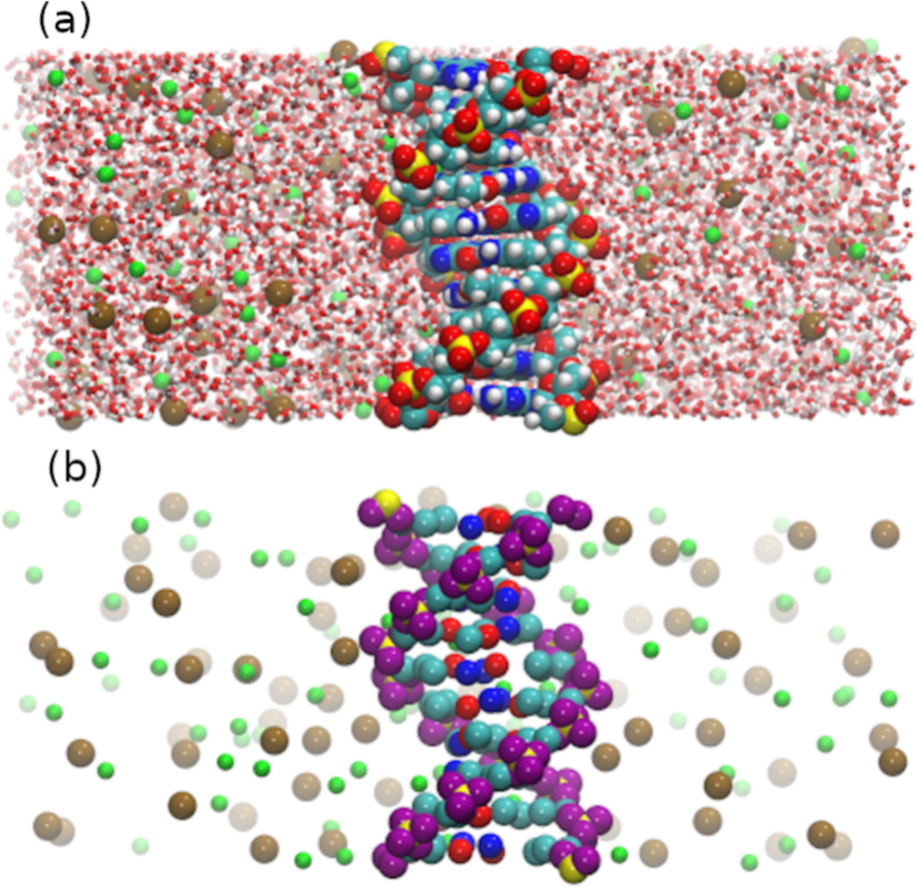
Cross section of the simulation box for simulations of a periodic DNA molecule at 1.0 mol L^–1^ sodium chloride salt solution. The sodium ions are shown in green, while the chloride ions are in ochre. (a) All-atom model with explicit solvation. The carbon, nitrogen, phosphate, oxygen, and hydrogen atoms are depicted in cyan, blue, yellow, red, and white, respectively. (b) DIS model with implicit solvation. Two types of oxygen atoms are colored red and purple. Both are explicitly defined in the prior model, but only the positions of the red oxygen atoms are used as input to the ML potential.

In the DIS model, the ions and the DNA molecule are treated explicitly, while the water is coarse-grained out (Figure 1). The DNA model is coarse-grained because the full all-atom description would not be computationally efficient for the ML potential of ions. To find the optimal level of coarse-graining, we explored different DNA representations in the literature.^109,110^ The selected CG mapping is based on the DNA model developed by Kovaleva et al.,^110^ where each nucleotide is represented by six CG sites. The CG model is sufficiently complex to enable the ML potential to correctly fit the effective interactions in the vicinity of the DNA.

The many-body potential of the mean force for the ions is composed of two parts: an ML potential and a prior potential. The ML potential is an ENN Allegro.^51^ It uses a strictly local many-body equivariant representation, resulting in excellent computational efficiency. For further information, the Allegro’s potential is described in Supporting Information and more in detail in developer’s papers.^51,111^ The parameters of ML model are trained with the force-matching approach,^112^ i.e., the training loss is defined as
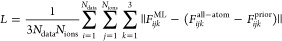
1where *F*_*ijk*_ is the force in the *k*-direction of the *j*-th ion in the *i*-th configuration. We use the Adam optimizer^113^ with the default parameters from pytorch.^114^ The details of the fit are reported in Supporting Information.

The prior potential prevents the exploration of physically invalid regions of the potential energy surface, such as particle overlaps.^64,115,116^ We find it to be especially important for small salt concentrations, i.e., low-density systems. Moreover, the ions, especially Na^+^ cations, exhibit numerous stable configurations within both the minor and major grooves.^15^ These configurations are already effectively captured by the prior model. The prior potential models the van der Waals and electrostatic interactions. For the former, we use the 12–6 Lennard-Jones potential with empirically optimized parameters (Supporting Information Table S1) to best fit the reference ion structural properties. For the latter, we employ the Coulomb interaction with the Wolf summation^117,118^ for the long-range correction. The nonbonded interactions are also calculated within cutoff distances of 0.9 and 1.2 nm, respectively, for the LJ and the electrostatic potential. Since water is treated implicitly as a dielectric continuum, the electrostatic interactions are screened. We use a dielectric constant of 95 as this value corresponds to the measured dielectric constant of the TIP3P water model.^119^ Additionally, to further improve the fit of the sodium-phosphate interaction, we added oxygen atoms bound to the phosphate atoms (Figure 1 purple). The added oxygen atoms improve the fit of the Na-phosphate first coordination shell. These atoms are explicitly modeled only with the Lennard-Jones potential; i.e., they have a zero charge and are not seen by the ML potential.

## 3 Computational Details

All simulations have been carried out using LAMMPS.^120^ Newton’s equations of motion are integrated using the Velocity Verlet integrator^121,122^ with a 1 fs time step. Simulations are performed at a temperature of 300 K. For all-atom simulations, we employ the Nosé–Hoover thermostat^123^ with a coupling constant of 0.1 ps. In the case of the *NPT* simulations maintained at 1.0 bar, we additionally use the Nosé–Hoover barostat with a coupling constant of 1.0 ps. For simulations employing the DIS model, we use the Langevin thermostat with a coupling constant of 0.1 ps. All simulations are performed under periodic boundary conditions. The cubic box edge is around 4.2 nm for the most concentrated solutions and 5.4 nm for the lowest one. For simulations including the DNA molecule, the box size is 8.5 × 8.5 × 3.4 nm, which corresponds to exactly one DNA pitch oriented in the *z*-direction. The periodic boundary conditions are also used for the DNA molecule as in previous studies.^124,125^ Thus, the DNA molecule is effectively infinitely long. To analyze the properties of the trained DIS model, we perform 50 ns *NVT* simulations after 1 ns equilibration for the pure salt solution systems. For DNA simulations, we perform a 25 ns *NVT* simulation after 1 ns equilibration.

## 4 Results and Discussion

### 4.1 Pure Ionic Aqueous Solutions

In the following, we compare our developed DIS model with the all-atom model, which serves as a target reference. Additionally, we show the results for the prior potential to highlight the inadequacy of simple classical potentials and the improvement made by the ML potential.

**Figure 2:**
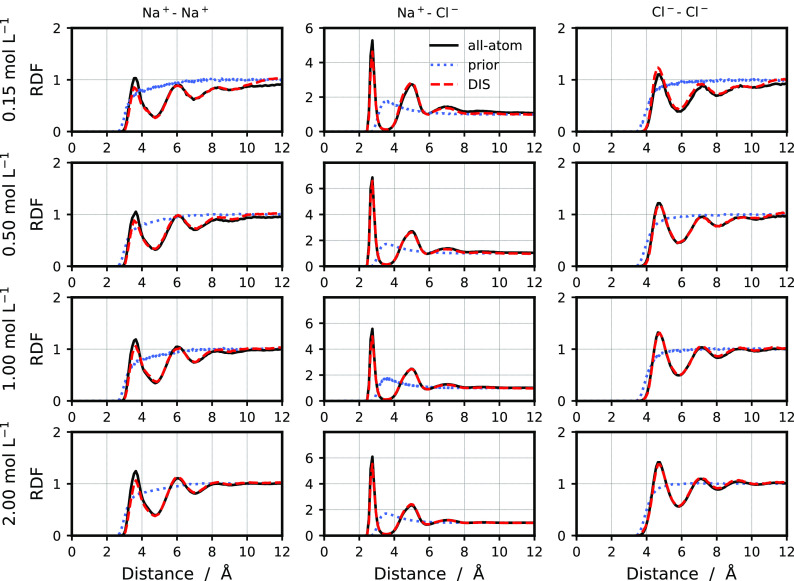
RDFs for Na^+^–Na^+^, Na^+^–Cl^–^, and Cl^–^–Cl^–^ pairs and concentrations 0.15, 0.5, 1.0, and 2.0 mol L^–1^. The all-atom, prior, and DIS models are colored black, blue, and red, respectively. The uncertainty in RDFs is too small to be seen.

We first consider the pure sodium chloride solution at four salt concentrations, 0.15, 0.5, 1.0, and 2.0 mol L^–1^. We investigate the structural properties by computing the radial distribution functions (RDFs) for the three ion–ion interactions, i.e., Na^+^–Na^+^, Na^+^–Cl^–^, and Cl^–^–Cl^–^ (Figure 2). As expected, we observe for all pairs that as concentration increases, the height of the first RDF’s peak increases with no shift in the position of the peak. The RDFs for the prior model exhibit ideal gas characteristics, i.e., a local structure highly dissimilar from the all-atom reference. With the addition of the ML potential, the effective ion–ion potentials are corrected and the RDFs are in agreement. The positions of the peaks are very well reproduced, while slight differences in the intensity of the peaks are observed for some RDFs. The differences are slightly higher at lower concentrations. The number of ions within the cutoff sphere of the ML potential is small at low concentrations. In particular, the coordination numbers, indicating how many ions can be found on average in a particular range, are shown in Supporting Information Figure S1. At the physiological salt concentration (0.15 mol L^–1^), the coordination numbers are smaller than one within the entire cutoff sphere. Consequently, these ML models are challenging to train. For the Na–Cl interaction, at each concentration, the solvent-separated pair exhibits two configurations at 4.6 and 5.0 Å. These are SSIP configurations already observed on RDF and confirmed by the calculation of the McMillan–Mayer potential.^126,127^ It is due to hydrogen bondings between the water molecules in the cation’s first coordination shell and the anion; it is more pronounced for other salts with a higher constant of association.

CG models are thermodynamically state dependent. In particular, they are salt concentration dependent. Thus, developing a transferable salt solution model that would be accurate at any arbitrary salt concentration is impossible. Nevertheless, we still expect that the models will be transferable for small salt concentration changes. We investigated this aspect by training the model at one specific concentration and testing it at all four concentrations. The corresponding RDFs are shown in Supporting Information Figures S2–S5. We observe a relatively good reproduction of structural properties for out-of-training salt concentrations. The exception is the DIS model trained at 0.15 mol L^–1^ and employed at the highest 2.0 mol L^–1^ concentration, where the results significantly deviate from the all-atom reference. As an additional test, we train the DIS model with configurations obtained from all-atom simulations of all concentrations. The hyperparameters were reoptimized in this case (see Supporting Information). As expected, the acquired structural properties (Supporting Information Figure S6) are less accurate than the models trained at a specific concentration, confirming the concentration dependency of the potential of the mean force. In the future, the salt concentration could be added as an additional input feature of the NN, making the model directly concentration-dependent. Similar approaches were previously proposed to include the temperature dependency of the CG models.^63^ To emphasize the importance of capturing the many-body term of the PMF, a pure sodium chloride solution at 5.0 mol kg^–1^ has been performed following the computational detail of Shen et al.,^83^ the detailed results are provided in the Supporting Information (Section 4.3). The DIS model and two other implicit models, i.e., the IBI potential^76,128^ fitted at 5.0 mol kg^–1^ and the transferable effective potential of the cited paper.^83^ The RDFs clearly highlight that addressing high-order atomistic correlations explicitly is essential for achieving a more accurate model.

The employed force-matching training strategy can theoretically reproduce all of the structural and thermodynamical properties of the underlying all-atom model. The dynamic properties, however, cannot be matched. CG models typically exhibit faster dynamics due to a smoother potential energy surface. Indeed, the average self-diffusion coefficient of ions, calculated via Einstein relation, for the all-atom and the DIS simulations 1.2 × 10^–9^ and 6.8 × 10^–9^ m^2^ s^–1^, respectively.

### 4.2 Periodic DNA in an Ionic Aqueous Solution

Having validated the DIS model for the aqueous NaCl salt solution, we introduced a periodic DNA molecule into the simulation box. The main challenge here is to accurately describe the ion interactions in different chemical environments, i.e., near the DNA molecule and bulk media. In the bulk, atom density is low and only composed of ions. In the vicinity of the DNA molecule, the density of the particles significantly increases. To be able to capture the effective interaction of the ions around the DNA molecule, the complexity of the atomistic geometry representation is increased, i.e., the number of Bessel functions, the sizes of the 2-body network and the high-order tensor are increasing. The ML potential details are reported in Supporting Information. We developed a DIS model for two salt concentrations. In the following, we discuss the 1.0 mol L^–1^ salt solution. The results at 0.5 mol L^–1^ salt concentration, reported in the Supporting Information, show the same tendency.

**Figure 3:**
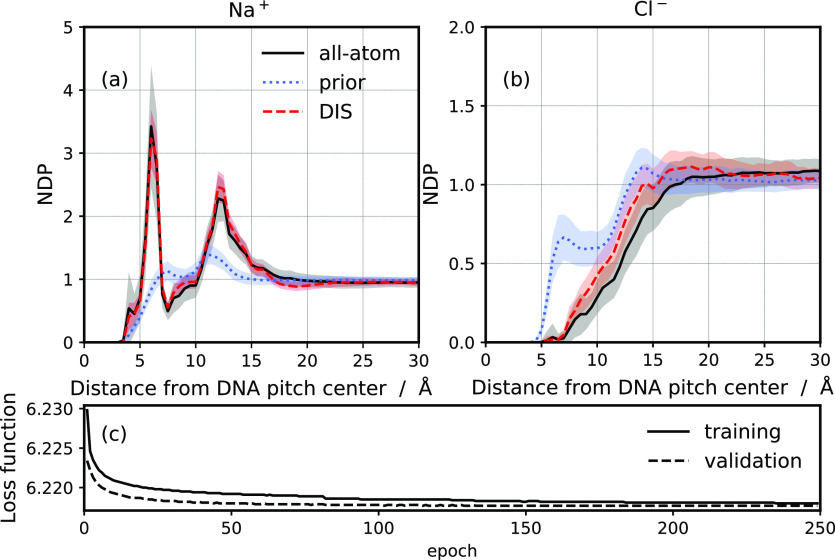
Cylindrical NDP for Na^+^ (a) and Cl^–^ (b) from the center-of-mass of the DNA molecule. The results are shown for the all-atom (black), prior (blue), and DIS (red) simulations at 1.0 mol L^–1^. The colored areas represent the standard deviation with a block averaging of 1 ns. The training (solid) and validation (dashed) loss functions as a function of the epochs (c).

First, we calculate the sodium and chloride cylindrical normalized density profiles (NDPs) from the center of mass of the DNA molecule (Figure 3). For Na^+^ ions, we obtain excellent agreement with all-atom simulations. For Cl^–^ ions, the density in the vicinity of the DNA molecule is slightly lower in the DIS model. Nonetheless, the structural properties are still significantly more accurate compared to the classical CG models employing explicit solvent modeling.^129,130^ This result demonstrates that the ML model can provide an accurate many-body potential of mean force even with the reduced DNA representation. Namely, the all-atom model contains 634 DNA atoms, while the input to the ML model is based on 120 explicit DNA atoms.

**Figure 4:**
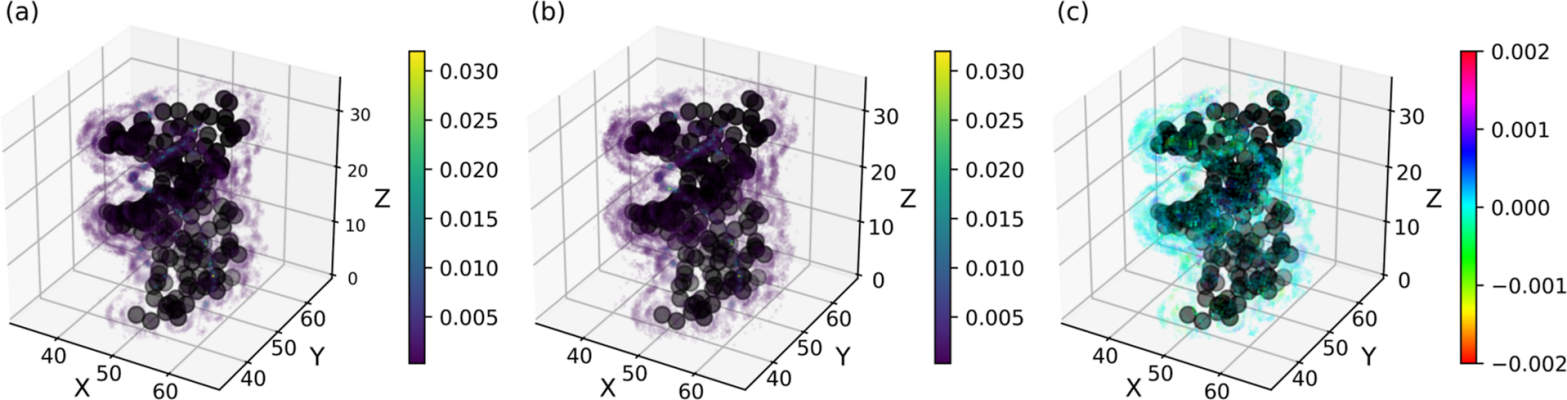
3D distribution of Na^+^ cations around the DNA pitch in a NaCl aqueous solution at 1.0 mol L^–1^ for the all-atom (a), DIS (b), and the difference between both simulations (c). The distribution is normalized over the trajectory and the total number of cations. For the sake of clarity, only the positions with a sufficient probability are represented. The black beads represent the CG resolution of the DNA molecule.

**Figure 5:**
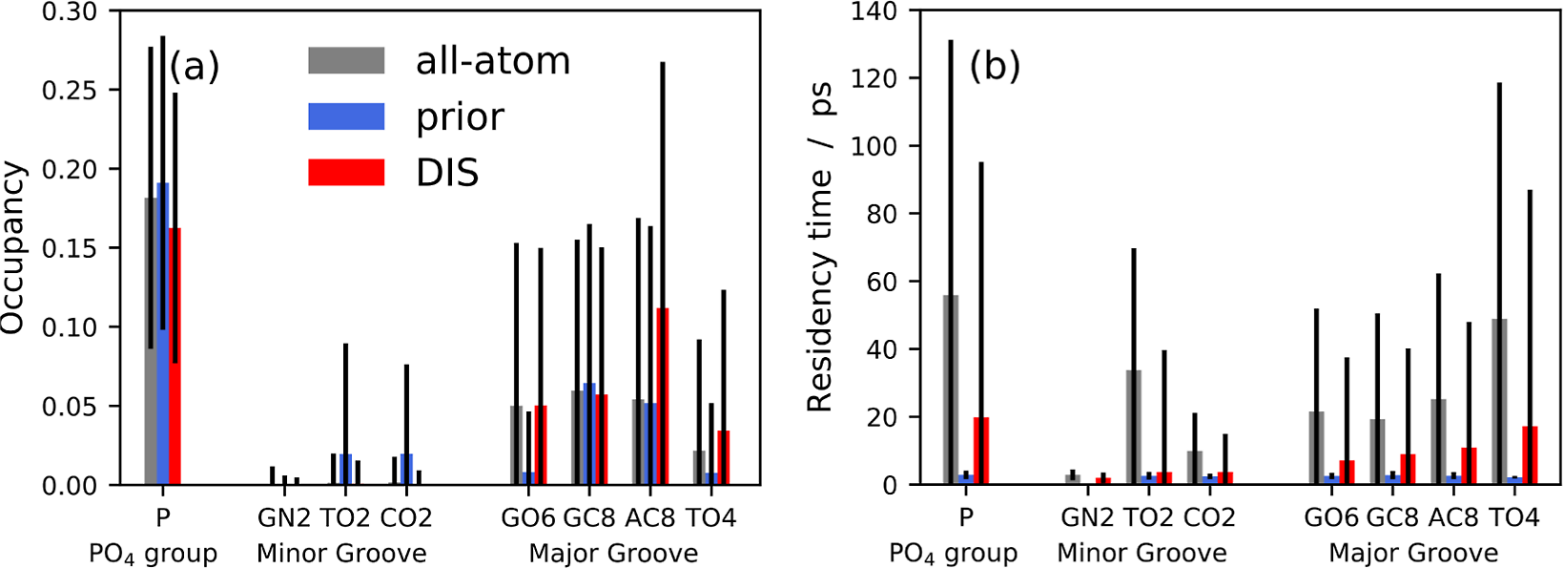
Average occupancy (a) and residence times (b) of Na^+^ ions in the first hydration shell of the atoms of DNA at 1.0 mol L^–1^. The error bars denote the standard deviation. The fast fluctuations (<1 ps) are omitted in the residence time calculation.

To visualize the ion distribution around the DNA molecule in 3D, we calculate the likelihood of observing Na^+^ ions at every grid point around the DNA molecule using a grid spacing of 0.5 Å. Despite a few discrepancies, Figure 4 confirms that the DIS model accurately describes the ion distribution. We computed the Na^+^ average occupancy in the first hydration shell and the associated residence times to further validate this point (Figure 5). The ion behavior in the DNA molecule’s backbone, minor, and major grooves is similar regarding the average occupancy. To enable a comparison between the all-atom and DIS models, we performed the calculation based on the CG DNA representation^110^ for both models. We observe a preferential binding of Na^+^ ions to the phosphodiester group and major groove, while the minor groove exhibits low occupancy. Similar findings were also previously reported.^124^ On the other hand, the all-atom and DIS models display differences in the residence times. In particular, the residence times for the DIS model are lower by an approximately constant factor of 2.8 for the phosphodiester group and 2.6 for the major groove. For the Minor groove, the range of values is between 1 and 9. Due to the faster dynamics of CG models, lower residence times are expected.^129^ However, as expected, the DIS model outperforms the prior model regarding the average and the standard deviation of the residence times. Indeed, the prior model gives a good first approximation of the potential of mean force regarding the average occupancy, but the local ion configurations around DNA are not described. Yet, Figures 3 and 5 confirm that the ML potential enables to description of the stable configurations of the ions in the vicinity of DNA and correctly approximate the many-body terms of the PMF.

For a fair comparison of simulation speedup, we run simulations on one CPU using the all-atom and the DIS models for two systems: pure salt solution at 0.15 mol L^–1^ and the DNA molecule embedded into a salt solution at 0.5 mol L^–1^. We use cutoff distances of 0.9 and 1.2 nm for computing the LJ and the electrostatic interactions, respectively, in both systems. For the pure salt solution, we obtain 0.5 and 84 ns per day for the all-atom model and the DIS MD, respectively. For the system containing the DNA molecule, we obtain 0.1 and 1.5 ns per day. The DIS model is competitive because of its much lower computational requirements and allows for simulating long trajectories as well as increasing the size of the system.

## 5 Conclusions

In conclusion, we present a new implicit solvation ML model of ionic media based on an equivariant graph neural network approach. Our DIS model showcased excellent accuracy with respect to the structural properties of aqueous salt solutions with concentrations ranging from 0.15 to 2.0 mol L^–1^. The molecular system including a DNA molecule likewise confirmed that the model can simultaneously capture the effective ion interaction in two distinct environments: close to the DNA molecule and in bulk. This work paves the way for efficient simulations of ionic media by using an implicit solvation model. Our approach is general and could be used with other ML potentials. This will allow us to go beyond the current size of the system to understand the complex effective interaction of biomolecules in ionic media.^131^ We will also consider other salt solutions with higher valency ions to understand the impact on the learning process and the complexity of the neural network.

MD simulations of DNA require an accurate incorporation of electrostatics into the model. We have used the delta-learning approach to derive the ML interaction potential where the prior model captures explicit long-range interactions using Wolf summation. This represents the main limitation of our ML model since explicit long-range electrostatics are lacking in the current model. As this might lead to structural artifacts, DNA is fixed in this study. This allows us to capture only the behavior of the ions alone. Nevertheless, such a model could already be useful to study solid–liquid interfaces,^132,133^ for example. Another limitation is the lack of transferability to other DNA sequences, as the model has been trained on only one specific DNA sequence and its corresponding fixed configuration. A cutoff-dependent Allegro potential could address this issue by using a large cutoff distance for ions and a shorter one for the DNA atoms and allow the introduction of a flexible DNA molecule in our model. This extension would enable the exploration of various CG mappings and their impact on the PMF in order to enhance the precision of the latter. An implementation of the prior model in Allegro’s framework could also be an improvement. Our future work will focus on extending our model along these lines.

## Supporting Information Available

Parameters of the prior model; description of the Allegro potential; details of the hyperparameters and training protocols for the machine learning potential; coordination number calculated from the RDFs of the Figure 2; RDFs for all the aqueous NaCl solutions with a deep potential trained at a specific concentration; RDFs for all the aqueous NaCl solutions with a deep potential trained with all concentration configurations; structural analyses of ions for a DNA molecule in an aqueous NaCl salt solution of 0.5 mol L^–1^; and comparison of pairwise and DIS potentials for a highly concentrated NaCl aqueous solution (PDF)

## References

[ref1] Šponer J.; Bussi G.; Krepl M.; Banáš P.; Bottaro S.; Cunha R. A.; Gil-Ley A.; Pinamonti G.; Poblete S.; Jurečka P.; Walter N. G.; Otyepka M. RNA Structural Dynamics As Captured by Molecular Simulations: A Comprehensive Overview. Chem. Rev. 2018, 118, 4177–4338. 10.1021/acs.chemrev.7b00427. 29297679 PMC5920944

[ref2] Calimet N.; Simoes M.; Changeux J.-P.; Karplus M.; Taly A.; Cecchini M. A gating mechanism of pentameric ligand-gated ion channels. Proc. Natl. Acad. Sci. U.S.A. 2013, 110, E3987–E3996. 10.1073/pnas.1313785110. 24043807 PMC3801054

[ref3] Pokorná P.; Krepl M.; Campagne S.; Šponer J. Conformational Heterogeneity of RNA Stem-Loop Hairpins Bound to FUS-RNA Recognition Motif with Disordered RGG Tail Revealed by Unbiased Molecular Dynamics Simulations. J. Phys. Chem. B 2022, 126, 9207–9221. 10.1021/acs.jpcb.2c06168. 36348631

[ref4] Workman R. J.; Gorle S.; Pettitt B. M. Effects of Conformational Constraint on Peptide Solubility Limits. J. Phys. Chem. B 2022, 126, 10510–10518. 10.1021/acs.jpcb.2c06458. 36450134 PMC10270293

[ref5] Sarthak K.; Winogradoff D.; Ge Y.; Myong S.; Aksimentiev A. Benchmarking Molecular Dynamics Force Fields for All-Atom Simulations of Biological Condensates. J. Chem. Theory Comput. 2023, 19, 3721–3740. 10.1021/acs.jctc.3c00148. 37134270 PMC11169342

[ref6] Barros E. P.; Schiffer J. M.; Vorobieva A.; Dou J.; Baker D.; Amaro R. E. Improving the Efficiency of Ligand-Binding Protein Design with Molecular Dynamics Simulations. J. Chem. Theory Comput. 2019, 15, 5703–5715. 10.1021/acs.jctc.9b00483. 31442033 PMC7532806

[ref7] Hirano Y.; Okimoto N.; Fujita S.; Taiji M. Molecular Dynamics Study of Conformational Changes of Tankyrase 2 Binding Subsites upon Ligand Binding. ACS Omega 2021, 6, 17609–17620. 10.1021/acsomega.1c02159. 34278146 PMC8280666

[ref8] Liu Y.; Prigozhin M. B.; Schulten K.; Gruebele M. Observation of Complete Pressure-Jump Protein Refolding in Molecular Dynamics Simulation and Experiment. J. Am. Chem. Soc. 2014, 136, 4265–4272. 10.1021/ja412639u. 24437525 PMC3985862

[ref9] Nassar R.; Brini E.; Parui S.; Liu C.; Dignon G. L.; Dill K. A. Accelerating Protein Folding Molecular Dynamics Using Inter-Residue Distances from Machine Learning Servers. J. Chem. Theory Comput. 2022, 18, 1929–1935. 10.1021/acs.jctc.1c00916. 35133832 PMC9281603

[ref10] Lindorff-Larsen K.; Piana S.; Palmo K.; Maragakis P.; Klepeis J. L.; Dror R. O.; Shaw D. E. Improved side-chain torsion potentials for the Amber ff99SB protein force field. Proteins: Struct., Funct., Bioinf. 2010, 78, 1950–1958. 10.1002/prot.22711. PMC2970904 20408171

[ref11] Bai G.; Pan Y.; Zhang Y.; Li Y.; Wang J.; Wang Y.; Teng W.; Jin G.; Geng F.; Cao J. Research advances of molecular docking and molecular dynamic simulation in recognizing interaction between muscle proteins and exogenous additives. Food Chem. 2023, 429, 136836 10.1016/j.foodchem.2023.136836. 37453331

[ref12] Wang J.; Alekseenko A.; Kozakov D.; Miao Y. Improved Modeling of Peptide-Protein Binding Through Global Docking and Accelerated Molecular Dynamics Simulations. Front. Mol. Biosci. 2019, 6, 112 10.3389/fmolb.2019.00112. 31737642 PMC6835073

[ref13] Cruz-León S.; Schwierz N. RNA Captures More Cations than DNA: Insights from Molecular Dynamics Simulations. J. Phys. Chem. B 2022, 126, 8646–8654. 10.1021/acs.jpcb.2c04488. 36260822 PMC9639116

[ref14] Karplus M.; McCammon J. A. Molecular dynamics simulations of biomolecules. Nat. Struct. Mol. Biol. 2002, 9, 646–652. 10.1038/nsb0902-646. 12198485

[ref15] Giambaşu G.; Luchko T.; Herschlag D.; York D.; Case D. Ion Counting from Explicit-Solvent Simulations and 3D-RISM. Biophys. J. 2014, 106, 883–894. 10.1016/j.bpj.2014.01.021. 24559991 PMC3944826

[ref16] Marinelli F.; Faraldo-Gómez J. Ensemble-Biased Metadynamics: A Molecular Simulation Method to Sample Experimental Distributions. Biophys. J. 2015, 108, 2779–2782. 10.1016/j.bpj.2015.05.024. 26083917 PMC4472218

[ref17] Wang J.; Ishchenko A.; Zhang W.; Razavi A.; Langley D. A highly accurate metadynamics-based Dissociation Free Energy method to calculate protein–protein and protein–ligand binding potencies. Sci. Rep. 2022, 12, 2024 10.1038/s41598-022-05875-8. 35132139 PMC8821539

[ref18] Meshkin H.; Zhu F. Thermodynamics of Protein Folding Studied by Umbrella Sampling along a Reaction Coordinate of Native Contacts. J. Chem. Theory Comput. 2017, 13, 2086–2097. 10.1021/acs.jctc.6b01171. 28355066

[ref19] You W.; Tang Z.; Chang C.-e. A. Potential Mean Force from Umbrella Sampling Simulations: What Can We Learn and What Is Missed?. J. Chem. Theory Comput. 2019, 15, 2433–2443. 10.1021/acs.jctc.8b01142. 30811931 PMC6456367

[ref20] Noid W. G. Perspective: Coarse-grained models for biomolecular systems. J. Chem. Phys. 2013, 139, 090901 10.1063/1.4818908. 24028092

[ref21] Riniker S.; Allison J. R.; van Gunsteren W. F. On developing coarse-grained models for biomolecular simulation: a review. Phys. Chem. Chem. Phys. 2012, 14, 12423–12430. 10.1039/c2cp40934h. 22678152

[ref22] Jin J.; Pak A. J.; Durumeric A. E. P.; Loose T. D.; Voth G. A. Bottom-up Coarse-Graining: Principles and Perspectives. J. Chem. Theory Comput. 2022, 18, 5759–5791. 10.1021/acs.jctc.2c00643. 36070494 PMC9558379

[ref23] Souza P. C. T.; Alessandri R.; Barnoud J.; Thallmair S.; Faustino I.; Grunewald F.; Patmanidis I.; Abdizadeh H.; Bruininks B. M. H.; Wassenaar T. A.; Kroon P. C.; Melcr J.; Nieto V.; Corradi V.; Khan H. M.; Domański J.; Javanainen M.; Martinez-Seara H.; Reuter N.; Best R. B.; Vattulainen I.; Monticelli L.; Periole X.; Tieleman D. P.; de Vries A. H.; Marrink S. J. Martini 3: a general purpose force field for coarse-grained molecular dynamics. Nat. Methods 2021, 18, 382–388. 10.1038/s41592-021-01098-3. 33782607 PMC12554258

[ref24] Balatti G. E.; Martini M. F.; Pickholz M. Investigating the Impact of the Glycolipid Content on Aurein 1.2 Pores in Prokaryotic Model Bilayers: A Coarse-Grain Molecular Dynamics Simulation Study. J. Phys. Chem. B 2023, 127, 5190–5198. 10.1021/acs.jpcb.3c01053. 37256556

[ref25] Visan R. M.; Angelescu D. G. Coarse-Grained Model of Phytic Acid for Predicting the Supramolecular Architecture of Ionically Cross-Linked Chitosan Hydrogels. J. Phys. Chem. B 2023, 127, 5718–5729. 10.1021/acs.jpcb.3c02115. 37253184

[ref26] Nguyen D. D.; Wang B.; Wei G.-W. Accurate, robust, and reliable calculations of Poisson–Boltzmann binding energies. J. Comput. Chem. 2017, 38, 941–948. 10.1002/jcc.24757. 28211071 PMC5844473

[ref27] Ringe S.; Oberhofer H.; Hille C.; Matera S.; Reuter K. Function-Space-Based Solution Scheme for the Size-Modified Poisson–Boltzmann Equation in Full-Potential DFT. J. Chem. Theory Comput. 2016, 12, 4052–4066. 10.1021/acs.jctc.6b00435. 27323006

[ref28] Lipparini F.; Mennucci B. Perspective: Polarizable continuum models for quantum-mechanical descriptions. J. Chem. Phys. 2016, 144, 160901 10.1063/1.4947236. 27131518

[ref29] Nguyen H.; Pérez A.; Bermeo S.; Simmerling C. Refinement of Generalized Born Implicit Solvation Parameters for Nucleic Acids and Their Complexes with Proteins. J. Chem. Theory Comput. 2015, 11, 3714–3728. 10.1021/acs.jctc.5b00271. 26574454 PMC4805114

[ref30] Tolokh I. S.; Thomas D. G.; Onufriev A. V. Explicit ions/implicit water generalized Born model for nucleic acids. J. Chem. Phys. 2018, 148, 195101 10.1063/1.5027260. 30307229 PMC5959738

[ref31] Collas P. Coulomb scattering in the Born approximation and the use of generalized functions. Am. J. Phys. 2021, 89, 799–805. 10.1119/10.0005453.

[ref32] Molina J. J.; Duvail M.; Guilbaud P.; Dufrêche J. F. Coarse-grained lanthanoid chloride aqueous solutions. J. Mol. Liq. 2010, 153, 107–111. 10.1016/j.molliq.2010.01.007.

[ref33] Gaillard T.; Case D. A. Evaluation of DNA force fields in implicit solvation. J. Chem. Theory Comput. 2011, 7, 3181–3198. 10.1021/ct200384r. 22043178 PMC3203201

[ref34] Nguyen H.; Perez A.; Bermeo S.; Simmerling C. Refinement of generalized born implicit solvation parameters for nucleic acids and their complexes with proteins. J. Chem. Theory Comput. 2015, 11, 3714–3728. 10.1021/acs.jctc.5b00271. 26574454 PMC4805114

[ref35] Praprotnik M.; Matysiak S.; Site L. D.; Kremer K.; Clementi C. Adaptive resolution simulation of liquid water. J. Phys.: Condens. Matter 2007, 19, 292201 10.1088/0953-8984/19/29/292201. 18205455

[ref36] Praprotnik M.; Site L. D.; Kremer K. Multiscale simulation of soft matter: From scale bridging to adaptive resolution. Annu. Rev. Phys. Chem. 2008, 59, 545–571. 10.1146/annurev.physchem.59.032607.093707. 18062769

[ref37] Matysiak S.; Clementi C.; Praprotnik M.; Kremer K.; Delle Site L. Modeling diffusive dynamics in adaptive resolution simulation of liquid water. J. Chem. Phys. 2008, 128, 024503 10.1063/1.2819486. 18205455

[ref38] Zavadlav J.; Podgornik R.; Praprotnik M. Adaptive resolution simulation of a DNA molecule in salt solution. J. Chem. Theory Comput. 2015, 11, 5035–5044. 10.1021/acs.jctc.5b00596. 26574288

[ref39] Zavadlav J.; Podgornik R.; Melo M. N.; Marrink S. J.; Praprotnik M. Adaptive resolution simulation of an atomistic DNA molecule in MARTINI salt solution. Eur. Phys. J.: Spec. Top. 2016, 225, 1595–1607. 10.1140/epjst/e2016-60117-8.

[ref40] Zavadlav J.; Sablić J.; Podgornik R.; Praprotnik M. Open-Boundary Molecular Dynamics of a DNA molecule in a hybrid explicit/implicit salt solution. Biophys. J. 2018, 114, 2352–2362. 10.1016/j.bpj.2018.02.042. 29650370 PMC6129463

[ref41] Zavadlav J.; Podgornik R.; Praprotnik M. Order and interactions in DNA arrays: Multiscale molecular dynamics simulation. Sci. Rep. 2017, 7, 4775 10.1038/s41598-017-05109-2. 28684875 PMC5500594

[ref42] Podgornik R.; Zavadlav J.; Praprotnik M. Molecular dynamics simulation of high density DNA arrays. Computation 2018, 6, 3 10.3390/computation6010003.

[ref43] Ceriotti M.; Clementi C.; Anatole von Lilienfeld O. Machine learning meets chemical physics. J. Chem. Phys. 2021, 154, 160401 10.1063/5.0051418. 33940847

[ref44] Behler J.; Parrinello M. Generalized Neural-Network Representation of High-Dimensional Potential-Energy Surfaces. Phys. Rev. Lett. 2007, 98, 146401 10.1103/PhysRevLett.98.146401. 17501293

[ref45] Kocer E.; Ko T. W.; Behler J. Neural Network Potentials: A Concise Overview of Methods. Annu. Rev. Phys. Chem. 2022, 73, 163–186. 10.1146/annurev-physchem-082720-034254. 34982580

[ref46] Unke O. T.; Chmiela S.; Sauceda H. E.; Gastegger M.; Poltavsky I.; Schütt K. T.; Tkatchenko A.; Müller K. R. Machine Learning Force Fields. Chem. Rev. 2021, 121, 10142–10186. 10.1021/acs.chemrev.0c01111. 33705118 PMC8391964

[ref47] Plé T.; Mauger N.; Adjoua O.; Inizan T. J.; Lagardère L.; Huppert S.; Piquemal J.-P. Routine Molecular Dynamics Simulations Including Nuclear Quantum Effects: From Force Fields to Machine Learning Potentials. J. Chem. Theory Comput. 2023, 19, 1432–1445. 10.1021/acs.jctc.2c01233. 36856658

[ref48] Noê F.; Tkatchenko A.; Müller K.; Clementi C. Machine Learning for Molecular Simulation. Annu. Rev. Phys. Chem. 2020, 71, 16 10.1146/annurev-physchem-042018-052331. 32092281

[ref49] Reiser P.; Neubert M.; Eberhard A.; Torresi L.; Zhou C.; Shao C.; Metni H.; van Hoesel C.; Schopmans H.; Sommer T.; Friederich P. Graph neural networks for materials science and chemistry. Commun. Mater. 2022, 3, 93 10.1038/s43246-022-00315-6. 36468086 PMC9702700

[ref50] Vandermause J.; Torrisi S. B.; Batzner S.; Xie Y.; Sun L.; Kolpak A. M.; Kozinsky B. On-the-fly active learning of interpretable Bayesian force fields for atomistic rare events. npj Comput. Mater. 2020, 6, 20 10.1038/s41524-020-0283-z.

[ref51] Musaelian A.; Batzner S.; Johansson A.; Sun L.; Owen C. J.; Kornbluth M.; Kozinsky B. Learning local equivariant representations for large-scale atomistic dynamics. Nat. Commun. 2023, 14, 579 10.1038/s41467-023-36329-y. 36737620 PMC9898554

[ref52] Batzner S.; Musaelian A.; Sun L.; Geiger M.; Mailoa J. P.; Kornbluth M.; Molinari N.; Smidt T. E.; Kozinsky B. E. (3)-equivariant graph neural networks for data-efficient and accurate interatomic potentials. Nat. Commun. 2022, 13, 2453 10.1038/s41467-022-29939-5. 35508450 PMC9068614

[ref53] Ko T. W.; Finkler J. A.; Goedecker S.; Behler J. A fourth-generation high-dimensional neural network potential with accurate electrostatics including non-local charge transfer. Nat. Commun. 2021, 12, 398 10.1038/s41467-020-20427-2. 33452239 PMC7811002

[ref54] Plé T.; Lagardère L.; Piquemal J.-P. Force-field-enhanced neural network interactions: from local equivariant embedding to atom-in-molecule properties and long-range effects. Chem. Sci. 2023, 14, 12554–12569. 10.1039/D3SC02581K. 38020379 PMC10646944

[ref55] Unke O. T.; Chmiela S.; Gastegger M.; Schuett K. T.; Sauceda H. E.; Mueller K.-R. SpookyNet: Learning force fields with electronic degrees of freedom and nonlocal effects. Nat. Commun. 2021, 12, 7273 10.1038/s41467-021-27504-0. 34907176 PMC8671403

[ref56] Zhang L.; Han J.; Wang H.; Car R. E. W.; E W. DeePCG: Constructing coarse-grained models via deep neural networks. J. Chem. Phys. 2018, 149, 034101 10.1063/1.5027645. 30037247

[ref57] Thaler S.; Zavadlav J. Learning neural network potentials from experimental data via Differentiable Trajectory Reweighting. Nat. Commun. 2021, 12, 6884 10.1038/s41467-021-27241-4. 34824254 PMC8617111

[ref58] Husic B. E.; Charron N. E.; Lemm D.; Wang J.; Pérez A.; Majewski M.; Krämer A.; Chen Y.; Olsson S.; de Fabritiis G.; Noé F.; Clementi C. Coarse graining molecular dynamics with graph neural networks. J. Chem. Phys. 2020, 153, 194101 10.1063/5.0026133. 33218238 PMC7671749

[ref59] Chen Y.; Krämer A.; Charron N. E.; Husic B. E.; Clementi C.; Noé F. Machine learning implicit solvation for molecular dynamics. J. Chem. Phys. 2021, 155, 084101 10.1063/5.0059915. 34470360

[ref60] Durumeric A. E.; Charron N. E.; Templeton C.; Musil F.; Bonneau K.; Pasos-Trejo A. S.; Chen Y.; Kelkar A.; Noé F.; Clementi C. Machine learned coarse-grained protein force-fields: Are we there yet?. Curr. Opin. Struct. Biol. 2023, 79, 102533 10.1016/j.sbi.2023.102533. 36731338 PMC10023382

[ref61] Katzberger P.; Riniker S. Implicit solvent approach based on generalized Born and transferable graph neural networks for molecular dynamics simulations. J. Chem. Phys. 2023, 158, 204101 10.1063/5.0147027. 37212404

[ref62] Thaler S.; Stupp M.; Zavadlav J. Deep Coarse-grained Potentials via Relative Entropy Minimization. J. Chem. Phys. 2022, 157, 244103 10.1063/5.0124538. 36586977

[ref63] Thaler S.; Doehner G.; Zavadlav J. Scalable Bayesian Uncertainty Quantification for Neural Network Potentials: Promise and Pitfalls. J. Chem. Theory Comput. 2023, 19, 4520–4532. 10.1021/acs.jctc.2c01267. 37014758

[ref64] Krämer A.; Durumeric A. E. P.; Charron N. E.; Chen Y.; Clementi C.; Noé F. Statistically Optimal Force Aggregation for Coarse-Graining Molecular Dynamics. J. Phys. Chem. Lett. 2023, 14, 3970–3979. 10.1021/acs.jpclett.3c00444. 37079800

[ref65] Vlachas P. R.; Zavadlav J.; Praprotnik M.; Koumoutsakos P. Accelerated Simulations of Molecular Systems through Learning of Effective Dynamics. J. Chem. Theory Comput. 2022, 18, 538–549. 10.1021/acs.jctc.1c00809. 34890204

[ref66] Wang S.; Ma Z.; Pan W. Data-Driven Coarse-Grained Modeling of Non-Equilibrium Systems. Soft Matter 2021, 17, 6404–6412. 10.1039/D1SM00413A. 34132317

[ref67] del Razo M. J.; Crommelin D.; Bolhuis P. G. Data-Driven Dynamical Coarse-Graining for Condensed Matter Systems. arXiv 2023, arXiv:2306.17672 10.48550/arXiv.2306.17672. 38193550

[ref68] Draper D. E.; Grilley D.; Soto A. M. Ions and RNA Folding. Annu. Rev. Biophys. 2005, 34, 221–243. 10.1146/annurev.biophys.34.040204.144511. 15869389

[ref69] Fingerhut B. P. The mutual interactions of RNA, counterions and water – quantifying the electrostatics at the phosphate–water interface. Chem. Commun. 2021, 57, 12880–12897. 10.1039/D1CC05367A. PMC8640580 34816825

[ref70] Nguyen H. T.; Hori N.; Thirumalai D. Theory and simulations for RNA folding in mixtures of monovalent and divalent cations. Proc. Natl. Acad. Sci. U.S.A. 2019, 116, 21022–21030. 10.1073/pnas.1911632116. 31570624 PMC6800359

[ref71] Hellström M.; Behler J. Structure of aqueous NaOH solutions: insights from neural-network-based molecular dynamics simulations. Phys. Chem. Chem. Phys. 2017, 19, 82–96. 10.1039/C6CP06547C. 27805193

[ref72] Hellström M.; Ceriotti M.; Behler J. Nuclear Quantum Effects in Sodium Hydroxide Solutions from Neural Network Molecular Dynamics Simulations. J. Phys. Chem. B 2018, 122, 10158–10171. 10.1021/acs.jpcb.8b06433. 30335385

[ref73] Schran C.; Thiemann F. L.; Rowe P.; Müller E. A.; Marsalek O.; Michaelides A. Machine learning potentials for complex aqueous systems made simple. Proc. Natl. Acad. Sci. U.S.A. 2021, 118, e2110077118 10.1073/pnas.2110077118. 34518232 PMC8463804

[ref74] Shao Y.; Hellström M.; Yllö A.; Mindemark J.; Hermansson K.; Behler J.; Zhang C. Temperature effects on the ionic conductivity in concentrated alkaline electrolyte solutions. Phys. Chem. Chem. Phys. 2020, 22, 10426–10430. 10.1039/C9CP06479F. 31895378

[ref75] Zhang J.; Pagotto J.; Duignan T. T. Towards predictive design of electrolyte solutions by accelerating ab initio simulation with neural networks. J. Mater. Chem. A 2022, 10, 19560–19571. 10.1039/D2TA02610D.

[ref76] Reith D.; Pütz M.; Müller-Plathe F. Deriving effective mesoscale potentials from atomistic simulations. J. Comput. Chem. 2003, 24, 1624–1636. 10.1002/jcc.10307. 12926006

[ref77] Potestio R.; Peter C.; Kremer K. Computer Simulations of Soft Matter: Linking the Scales. Entropy 2014, 16, 4199–4245. 10.3390/e16084199.

[ref78] Izvekov S.; Voth G. A. A Multiscale Coarse-Graining Method for Biomolecular Systems. J. Phys. Chem. B 2005, 109, 2469–2473. 10.1021/jp044629q. 16851243

[ref79] Wang Y.; Noid W. G.; Liu P.; Voth G. A. Effective force coarse-graining. Phys. Chem. Chem. Phys. 2009, 11, 2002–2015. 10.1039/b819182d. 19280011

[ref80] Lu L.; Dama J. F.; Voth G. A. Fitting coarse-grained distribution functions through an iterative force-matching method. J. Chem. Phys. 2013, 139, 121906 10.1063/1.4811667. 24089718

[ref81] Shell M. S. The relative entropy is fundamental to multiscale and inverse thermodynamic problems. J. Chem. Phys. 2008, 129, 144108 10.1063/1.2992060. 19045135

[ref82] Hess B.; Holm C.; van der Vegt N. Osmotic coefficients of atomistic NaCl (aq) force fields. J. Chem. Phys. 2006, 124, 164509 10.1063/1.2185105. 16674148

[ref83] Shen J.-W.; Li C.; van der Vegt N. F.; Peter C. Transferability of Coarse Grained Potentials: Implicit Solvent Models for Hydrated Ions. J. Chem. Theory Comput. 2011, 7, 1916–1927. 10.1021/ct2001396. 26596452

[ref84] Scherer C.; Andrienko D. Understanding three-body contributions to coarse-grained force fields. Phys. Chem. Chem. Phys. 2018, 20, 22387–22394. 10.1039/C8CP00746B. 30129962

[ref85] Wang J.; Olsson S.; Wehmeyer C.; Perez A.; Charron N. E.; de Fabritiis G.; Noe F.; Clementi C. Machine Learning of Coarse-Grained Molecular Dynamics Force Fields. ACS Cent. Sci. 2019, 5, 755–767. 10.1021/acscentsci.8b00913. 31139712 PMC6535777

[ref86] Wang J.; Charron N.; Husic B.; Olsson S.; Noé F.; Clementi C. Multi-body effects in a coarse-grained protein force field. J. Chem. Phys. 2021, 154, 164113 10.1063/5.0041022. 33940848

[ref87] Gkeka P.; Stoltz G.; Barati Farimani A.; Belkacemi Z.; Ceriotti M.; Chodera J. D.; Dinner A. R.; Ferguson A. L.; Maillet J.-B.; Minoux H.; Peter C.; Pietrucci F.; Silveira A.; Tkatchenko A.; Trstanova Z.; Wiewiora R.; Lelièvre T. Machine Learning Force Fields and Coarse-Grained Variables in Molecular Dynamics: Application to Materials and Biological Systems. J. Chem. Theory Comput. 2020, 16, 4757–4775. 10.1021/acs.jctc.0c00355. 32559068 PMC8312194

[ref88] Noid W. G. Perspective: Advances, Challenges, and Insight for Predictive Coarse-Grained Models. J. Phys. Chem. B 2023, 127, 4174–4207. 10.1021/acs.jpcb.2c08731. 37149781

[ref89] Sagui C.; Darden T. A. Molecular Dynamics Simulations of Biomolecules: Long-Range Electrostatic Effects. Annu. Rev. Biophys. Biomol. Struct. 1999, 28, 155–179. 10.1146/annurev.biophys.28.1.155. 10410799

[ref90] Li J.; Wijeratne S. S.; Qiu X.; Kiang C.-H. DNA under Force: Mechanics, Electrostatics, and Hydration. Nanomaterials 2015, 5, 246–267. 10.3390/nano5010246. 28347009 PMC5312857

[ref91] Cheng B.; Engel E. A.; Behler J.; Dellago C.; Ceriotti M. Ab initio thermodynamics of liquid and solid water. Proc. Natl. Acad. Sci. U.S.A. 2019, 116, 1110–1115. 10.1073/pnas.1815117116. 30610171 PMC6347673

[ref92] Staacke C. G.; Heenen H. H.; Scheurer C.; Csányi G.; Reuter K.; Margraf J. T. On the Role of Long-Range Electrostatics in Machine-Learned Interatomic Potentials for Complex Battery Materials. ACS Appl. Energy Mater. 2021, 4, 12562–12569. 10.1021/acsaem.1c02363.

[ref93] Vondrák M.; Reuter K.; Margraf J. T. q-pac: A Python package for machine learned charge equilibration models. J. Chem. Phys. 2023, 159, 054109 10.1063/5.0156290. 37530116

[ref94] Grisafi A.; Ceriotti M. Incorporating long-range physics in atomic-scale machine learning. J. Chem. Phys. 2019, 151, 204105 10.1063/1.5128375. 31779318

[ref95] Ko T. W.; Finkler J. A.; Goedecker S.; Behler J. Accurate Fourth-Generation Machine Learning Potentials by Electrostatic Embedding. J. Chem. Theory Comput. 2023, 19, 3567–3579. 10.1021/acs.jctc.2c01146. 37289440

[ref96] Deng Z.; Chen C.; Li X.-G.; Ong S. P. An electrostatic spectral neighbor analysis potential for lithium nitride. npj Comput. Mater. 2019, 5, 75 10.1038/s41524-019-0212-1.

[ref97] Wengert S.; Csányi G.; Reuter K.; Margraf J. T. Data-efficient machine learning for molecular crystal structure prediction. Chem. Sci. 2021, 12, 4536–4546. 10.1039/D0SC05765G. 34163719 PMC8179468

[ref98] Wengert S.; Csányi G.; Reuter K.; Margraf J. T. A Hybrid Machine Learning Approach for Structure Stability Prediction in Molecular Co-crystal Screenings. J. Chem. Theory Comput. 2022, 18, 4586–4593. 10.1021/acs.jctc.2c00343. 35709378 PMC9281391

[ref99] Batatia I.; Batzner S. L.; Kovács D. P.; Musaelian A.; Simm G. N. C.; Drautz R.; Ortner C.; Kozinsky B.; Csányi G. The Design Space of E(3)-Equivariant Atom-Centered Interatomic Potentials. arXiv 2022, arXiv:2205.06643 preprint 10.48550/arXiv.2205.0664web3. PMC11769842 39877429

[ref100] Stocker S.; Gasteiger J.; Becker F.; Günnemann S.; Margraf J. T. How robust are modern graph neural network potentials in long and hot molecular dynamics simulations?. Machine Learning: Science and Technology; IOP, 2022; Vol. 3, p 045010.

[ref101] Ramakrishnan R.; Dral P. O.; Rupp M.; Von Lilienfeld O. A. Big data meets quantum chemistry approximations: the Δ-machine learning approach. J. Chem. Theory Comput. 2015, 11, 2087–2096. 10.1021/acs.jctc.5b00099. 26574412

[ref102] Gao A.; Remsing R. C. Self-consistent determination of long-range electrostatics in neural network potentials. Nat. Commun. 2022, 13, 1572 10.1038/s41467-022-29243-2. 35322046 PMC8943018

[ref103] Anstine D. M.; Isayev O. Machine Learning Interatomic Potentials and Long-Range Physics. J. Phys. Chem. A 2023, 127, 2417–2431. 10.1021/acs.jpca.2c06778. 36802360 PMC10041642

[ref104] Jorgensen W. L.; Chandrasekhar J.; Madura J. D.; Impey R. W.; Klein M. L. Comparison of simple potential functions for simulating liquid water. J. Chem. Phys. 1983, 79, 926–935. 10.1063/1.445869.

[ref105] Duan Y.; Wu C.; Chowdhury S.; Lee M.; Xiong G.; Zhang W.; Yang R.; Cieplak P.; Luo R.; Lee T.; Caldwell J.; Wang J.; Kollman P. A point-charge force field for molecular mechanics simulations of proteins based on condensed-phase quantum mechanical calculations. J. Comput. Chem. 2003, 24, 1999–2012. 10.1002/jcc.10349. 14531054

[ref106] Joung I. S.; Cheatham T. E. I. Determination of Alkali and Halide Monovalent Ion Parameters for Use in Explicitly Solvated Biomolecular Simulations. J. Phys. Chem. B 2008, 112, 9020–9041. 10.1021/jp8001614. 18593145 PMC2652252

[ref107] Yoo J.; Aksimentiev A. Improved Parametrization of Li+, Na+, K+, and Mg2+ Ions for All-Atom Molecular Dynamics Simulations of Nucleic Acid Systems. J. Phys. Chem. Lett. 2012, 3, 45–50. 10.1021/jz201501a.

[ref108] Eastwood J.; Hockney R.; Lawrence D. P3M3DP—The three-dimensional periodic particle-particle/particle-mesh program. Comput. Phys. Commun. 1980, 19, 215–261. 10.1016/0010-4655(80)90052-1.

[ref109] Sun T.; Minhas V.; Korolev N.; Mirzoev A.; Lyubartsev A. P.; Nordenskiöld L. Bottom-Up Coarse-Grained Modeling of DNA. Front. Mol. Biosci. 2021, 8, 159 10.3389/fmolb.2021.645527. PMC8010198 33816559

[ref110] Kovaleva N. A.; Kikot I. P. K.; Mazo M. A.; Zubova E. A. The “sugar” coarse-grained DNA model. Journal of Molecular Modeling; Springer, 2014; Vol. 23, pp 1–16. 10.1007/s00894-017-3209-z 28185115

[ref111] Musaelian A.; Johansson A.; Batzner S.; Kozinsky B. Scaling the leading accuracy of deep equivariant models to biomolecular simulations of realistic size. arXiv 2023, arXiv:2304.10061 preprint 10.48550/arXiv.2304.1006web1.

[ref112] Noid W. G.; Chu J.-W.; Ayton G. S.; Krishna V.; Izvekov S.; Voth G. A.; Das A.; Andersen H. C. The multiscale coarse-graining method. I. A rigorous bridge between atomistic and coarse-grained models. J. Chem. Phys. 2008, 128, 244114 10.1063/1.2938860. 18601324 PMC2671183

[ref113] Kingma D. P.; Ba J. Adam: A Method for Stochastic Optimization. arXiv 2014, arXiv:1412.6980 10.48550/arXiv.1412.6980.

[ref114] Paszke A.; Gross S.; Massa F.; Lerer A.; Bradbury J.; Chanan G.; Killeen T.; Lin Z.; Gimelshein N.; Antiga L.; Desmaison A.; Kopf A.; Yang E.; DeVito Z.; Raison M.; Tejani A.; Chilamkurthy S.; Steiner B.; Fang L.; Bai J.; Chintala S. PyTorch: An Imperative Style, High-Performance Deep Learning Library. Adv. Neural Inf. Process. 2019, 32, 8024–8035.

[ref115] Morrow J. D.; Gardner J. L. A.; Deringer V. L. How to validate machine-learned interatomic potentials. J. Chem. Phys. 2023, 158, 121501 10.1063/5.0139611. 37003727

[ref116] Goodlett S. M.; Turney J. M.; Schaefer H. F.; Henry F. Comparison of multifidelity machine learning models for potential energy surfaces. J. Chem. Phys. 2023, 159, 044111 10.1063/5.0158919. 37493132

[ref117] Wolf D.; Keblinski P.; Phillpot S. R.; Eggebrecht J. Exact method for the simulation of Coulombic systems by spherically truncated, pairwise r-1 summation. J. Chem. Phys. 1999, 110, 8254–8282. 10.1063/1.478738.

[ref118] Gdoutos E. E.; Agrawal R.; Espinosa H. D. Comparison of the Ewald and Wolf methods for modeling electrostatic interactions in nanowires. Int. J. Numer Methods Eng. 2010, 84, 1541–1551. 10.1002/nme.2948.

[ref119] Kadaoluwa Pathirannahalage S. P.; Meftahi N.; Elbourne A.; Weiss A. C. G.; McConville C. F.; Padua A.; Winkler D. A.; Costa Gomes M.; Greaves T. L.; Le T. C.; Besford Q. A.; Christofferson A. J. Systematic Comparison of the Structural and Dynamic Properties of Commonly Used Water Models for Molecular Dynamics Simulations. J. Chem. Inf. Model. 2021, 61, 4521–4536. 10.1021/acs.jcim.1c00794. 34406000

[ref120] Thompson A. P.; Aktulga H. M.; Berger R.; Bolintineanu D. S.; Brown W. M.; Crozier P. S.; in t Veld P. J.; Kohlmeyer A.; Moore S. G.; Nguyen T. D.; Shan R.; Stevens M. J.; Tranchida J.; Trott C.; Plimpton S. J. LAMMPS - a flexible simulation tool for particle-based materials modeling at the atomic, meso, and continuum scales. Comput. Phys. Commun. 2022, 271, 108171 10.1016/j.cpc.2021.108171.

[ref121] Verlet L.; Levesque D. On the Theory of Classical Fluids II. Physica 1962, 28, 1124–1142. 10.1016/0031-8914(62)90058-7.

[ref122] Verlet L. Computer “Experiments” on Classical Fluids. I. Thermodynamical Properties of Lennard-Jones Molecules. Phys. Rev. 1967, 159, 98–103. 10.1103/PhysRev.159.98.

[ref123] Evans D. J.; Holian B. L. The Nose–Hoover thermostat. J. Phys. Chem. 1985, 83, 4069–4074. 10.1063/1.449071.

[ref124] Zavadlav J.; Podgornik R.; Praprotnik M. Adaptive Resolution Simulation of a DNA Molecule in Salt Solution. J. Chem. Theory Comput. 2015, 11, 5035–5044. 10.1021/acs.jctc.5b00596. 26574288

[ref125] Zavadlav J.; Sablić J.; Podgornik R.; Praprotnik M. Open-Boundary Molecular Dynamics of a DNA Molecule in a Hybrid Explicit/Implicit Salt Solution. Biophys. J. 2018, 114, 2352–2362. 10.1016/j.bpj.2018.02.042. 29650370 PMC6129463

[ref126] Duvail M.; Villard A.; Nguyen T.-N.; Dufrêche J. F. Thermodynamics of Associated Electrolytes in Water: Molecular Dynamics Simulations of Sulfate Solutions. J. Phys. Chem. B 2015, 119, 11184–11195. 10.1021/acs.jpcb.5b03088. 25965186

[ref127] Coste A.; Poulesquen A.; Diat O.; Dufrêche J. F.; Duvail M. Investigation of the Structure of Concentrated NaOH Aqueous Solutions by Combining Molecular Dynamics and Wide-Angle X-ray Scattering. J. Phys. Chem. B 2019, 123, 5121–5130. 10.1021/acs.jpcb.9b00495. 31141363

[ref128] Bevc S.; Junghans C.; Praprotnik M. STOCK: Structure mapper and online coarse-graining kit for molecular simulations. J. Comput. Chem. 2015, 36, 467–477. 10.1002/jcc.23806. 25504076

[ref129] DeMille R. C.; Cheatham T. E. I.; Molinero V. A Coarse-Grained Model of DNA with Explicit Solvation by Water and Ions. J. Phys. Chem. B 2011, 115, 132–142. 10.1021/jp107028n. 21155552 PMC3019136

[ref130] Uusitalo J. J.; Ingólfsson H. I.; Akhshi P.; Tieleman D. P.; Marrink S. J. Martini coarse-grained force field: extension to DNA. J. Chem. Theory Comput. 2015, 11, 3932–3945. 10.1021/acs.jctc.5b00286. 26574472

[ref131] Alexiou T. S.; Likos C. N. Effective Interactions between Double-Stranded DNA Molecules in Aqueous Electrolyte Solutions: Effects of Molecular Architecture and Counterion Valency. J. Phys. Chem. B 2023, 127, 6969–6981. 10.1021/acs.jpcb.3c02216. 37493448 PMC10424236

[ref132] Willemsen J. A. R.; Myneni S. C. B.; Bourg I. C. Molecular Dynamics Simulations of the Adsorption of Phthalate Esters on Smectite Clay Surfaces. J. Phys. Chem. C 2019, 123, 13624–13636. 10.1021/acs.jpcc.9b01864.

[ref133] Scalfi L.; Salanne M.; Rotenberg B. Molecular Simulation of Electrode-Solution Interfaces. Annu. Rev. Phys. Chem. 2021, 72, 189–212. 10.1146/annurev-physchem-090519-024042. 33395545

