# Supplementary material for: Developing an Implicit Solvation Machine Learning Model for Molecular Simulations of Ionic Media
Source: J Chem Theory Comput. 2023 Dec 20;20(1):411–20. doi: 10.1021/acs.jctc.3c00984 (PMC10782447; doi:10.1021/acs.jctc.3c00984)
Supplement: Supplementary file 1 — ct3c00984_si_001.pdf [file ct3c00984_si_001.pdf]

Supporting Information: Developing an Implicit Solvation Machine Learning Model for Molecular Simulations of Ionic Media

Amaury Coste,[†] Ema Slejko,[†,¶] Julija Zavadlav,[‡] and Matej Praprotnik[*,†,¶]

†*Laboratory for Molecular Modeling, National Institute of Chemistry, Ljubljana SI-1001, Slovenia;*

‡*Professorship of Multiscale Modeling of Fluid Materials, TUM School of Engineering and Design, Technical University of Munich, DE-85748 Garching near Munich, Germany*

¶*Department of Physics, Faculty of Mathematics and Physics, University of Ljubljana, Ljubljana SI-1000, Slovenia*

E-mail: praprot@cmm.ki.si

1 Prior model

The parameters of the 12-6 Lennard-Jones (LJ) potential are reported in Table S1. The atomic partial charges for the electrostatic interaction are +1 and -1 for the Na^+ and Cl^- ions, respectively. For the coarse-grained representation of the DNA, the atomic partial charges are defined in Ref.[1]

2 Allegro potential

The atomistic structures are represented by collections of nodes and edges. In the graph representation, the nodes correspond to atoms and edges connect the atom to each other

Table S1: 12-6 Lennard-Jones parameters for the prior model.

Atom i	Atom j	ϵ (kcal mol L^{-1})	σ (Å)
Na	Na	0.08744	3.20000
	Cl	0.05578	3.45847
	C	0.08672	3.10000
	N	0.1200	3.80000
	O	0.08718	2.45000
	P	0.13221	3.09050
Cl	Cl	0.03559	4.48000
	C	0.0553	3.9000
	N	0.07778	3.9000
	O	0.05562	4.0000
	P	0.08434	4.4000

within a cutoff-distance sphere. Allegro's potential is a strictly local equivariant deep neural network interatomic potential architecture with a graph representation.[2] In Allegro, the total predicted energy E_{system} of the system is decomposed into atomic energies E_i

$$E_{\text{system}} = \sum_{i}^{N} \sigma_{Z_i} E_i + \mu_{Z_i} \tag{S1}$$

where σ_{Z_i} and μ_{Z_i} are respectively a per-species scale and a shift parameter. The atomic energy E_i is also decomposed into a sum of pairwise energy E_{ij}

$$E_i = \sum_{j \in \mathcal{N}(i)} E_{ij} \tag{S2}$$

where $\mathcal{N}(i)$ is the local environment of atom i. By definition, E_{ij} is different from E_{ji} . The predicted force applied on atom i is then the negative gradient of the total energy with respect to the position of atom i:

$$\vec{F}_i = -\nabla E_{\text{system}} \tag{S3}$$

The Atomic Cluster Expansion (ACE) is used to describe the local atomic environment in a body-ordered expansion. It firstly project the local atomic density onto a set of radial bessel and spherical harmonic angular basis functions.

The Allegro's equivariant features $\mathbf{V}^{i,j,L}_{n,l,p}$ are indexed by an ordered pair of neighboring atoms (i, j) at each layer L. The features can be expressed as a direct sum of irreducible representations ("irreps") of the $O(3)$ rotation and mirror symmetry group, which are characterized by a rotation order l ranging from 0 and upward and parity p equal to ± 1. These features consist of scalars ($l = 0$), vectors ($l = 1$), and higher-order geometric tensors ($l \geq 2$). The index n corresponds to an additional feature channel. Allegro's potential defines the updated equivariant features on the pair ij as a weighted sum of the tensor products of the current features with the geometry of the other neighbor pairs ik in the local environment of i:

$$\mathbf{V}^{ij,L}_{n,(\ell_1,p_1,\ell_2,p_2)\to(\ell_{\mathrm{out}}\ ,p_{\mathrm{out}}\ )} = \mathbf{V}^{ij,L-1}_{n,\ell_1,p_1} \otimes \left(\sum_{k\in\mathcal{N}(i)} w^{ik,L}_{n,\ell_2,p_2} \vec{Y}^{ik}_{\ell_2,p_2} \right) \tag{S4}$$

3 Hyperparameters for MLP

Aqueous NaCl salt solutions trained at a specific concentration

We first investigated the cutoff distance hyperparameter and tested values from 8 to 12 Å. If the cutoff distance was set too small, we observed unphysical trajectories, e.g., phase separation of ions. The reason is that small cutoffs lead to a low number of neighbors seen by the ML potential, which makes the learning process harder for small cutoffs. Thus, the cutoff was set to 12 Å. We set the trainable Bessel functions to 12 with a cutoff polynomial envelope function using $p = 24$ after testing values between 8 and 12. The number of Bessel functions does not impact the results significantly. We found one layer with 8 features with an even parity to be sufficient. The parameter $l_{\max}$ is 2, increasing the value did not improve the fit. A 2-body latent multi-layer perceptron consists of 2 hidden layers of dimensions [16,

32] with a SILU non-linearity that is used for all the concentrations. The final edge energy multi-layer perceptron has one hidden layer of 16. The same Allegro architecture can be used for all concentrations, simplifying the hyperparameter studies. In general, the variations in Allegro-related hyperparameters led to small differences in the RDF peak heights but did not drastically change the structural properties. We also observed similar validation loss for these variations. For all the concentrations, the learning rate is set to 0.002 and reduced using an on-plateau scheduler based on the validation loss with a patience of 20 and a decay factor of 0.5. The batch size values, from 0.15 to 2.0 mol L^{-1}, are 16, 10, 10, and 5.

Aqueous NaCl salt solutions trained with all the concentrations

The hyperparameters that were used to train the model at a specific concentration did not give successful results when training on all salt concentration configurations. Thus, we increased the complexity of the Allegro network. We tried using a 2-body latent multi-layer perceptron consisting of 2 hidden layers of dimensions [32, 64], two layers with 8 features with an even parity, and one layer with 16 features with an even parity. We also tested the batch sizes of 32, 64, and 128. We obtained the best results with a batch size of 64. For all the training, the learning rate is set to 0.002 and reduced using an on-plateau scheduler based on the validation loss with a patience of 20 and a decay factor of 0.5.

Aqueous NaCl salt solutions in the presence of a DNA molecule

An increased complexity of the Allegro network compared to pure salt solution models was necessary. We set the number of trainable Bessel functions to 16 with a cutoff polynomial envelope function using $p = 24$. We use one layer with 16 features with an even parity. A 2-body latent multi-layer perceptron consists of 2 hidden layers of dimensions [32, 64] with a SILU non-linearity. The hidden layer dimension of the latent multi-layer perceptron is tripled. The final edge energy multi-layer perceptron has one hidden layer of 32. The

batch size is 16. For both trainings, the learning rate is set to 0.001 and reduced using an on-plateau scheduler based on the validation loss with a patience of 50 and a decay factor of 0.5.

4 Additional Results

4.1 Pure aqueous NaCl salt solutions

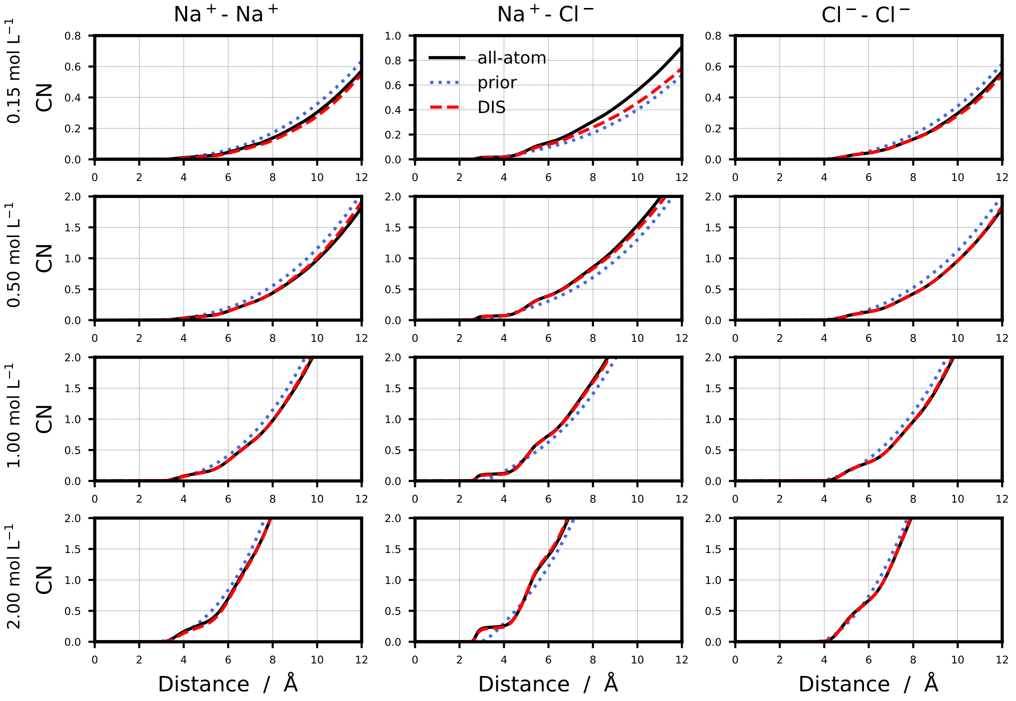

Figure S1: Coordination Number (CN) calculated from the radial distribution functions for the Na^+–Na^+, Na^+–Cl^- and Cl^-–Cl^- pairs and concentrations 0.15, 0.5, 1.0 and 2.0 mol L^{-1}. The all-atom, prior, and DIS models are shown in black, blue, and red, respectively.

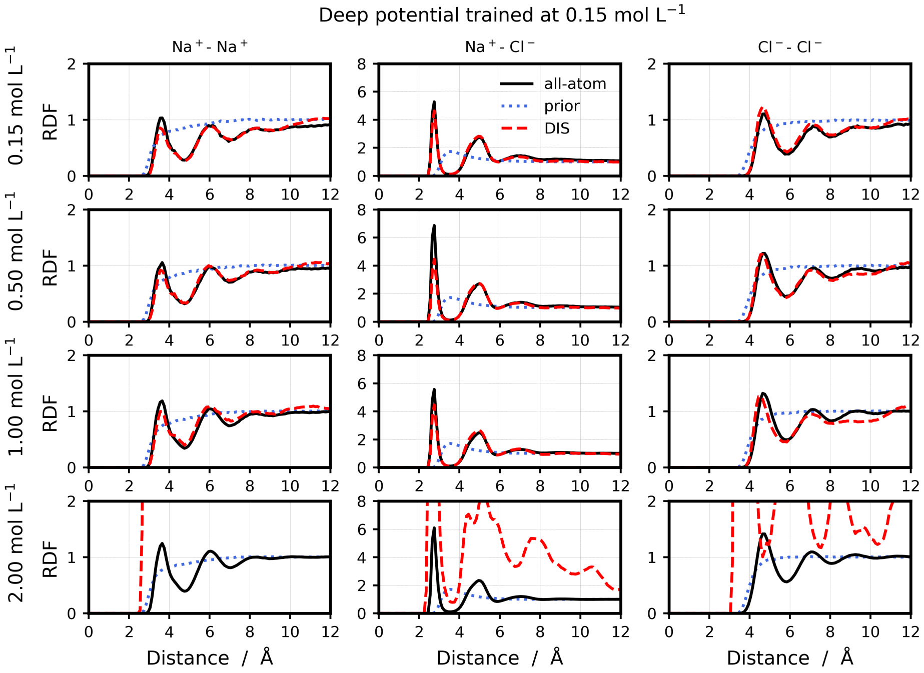

Figure S2: Radial Distribution Functions (RDFs) for the Na^+–Na^+, Na^+–Cl^- and Cl^-–Cl^- pairs and concentrations 0.15, 0.5, 1.0 and 2.0 mol L^{-1}. The deep potential has been trained at 0.15 mol L^{-1}. The all-atom, prior, and DIS models are shown in black, blue, and red, respectively.

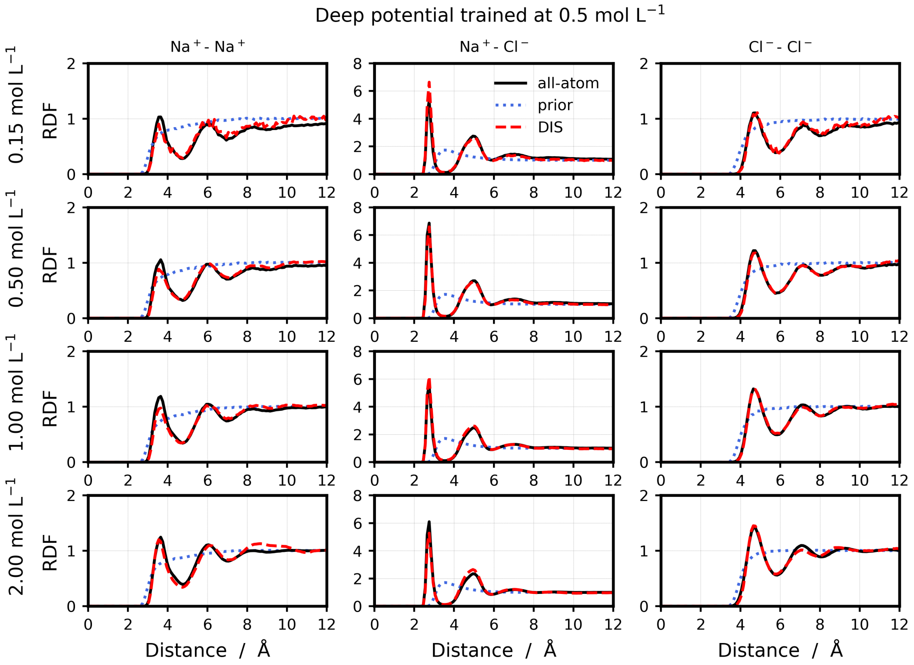

Figure S3: Radial Distribution Functions (RDFs) for the Na^+–Na^+, Na^+–Cl^- and Cl^-–Cl^- pairs and concentrations 0.15, 0.5, 1.0 and 2.0 mol L^{-1}. The deep potential has been trained at 0.5 mol L^{-1}. The all-atom, prior, and DIS models are shown in black, blue, and red, respectively.

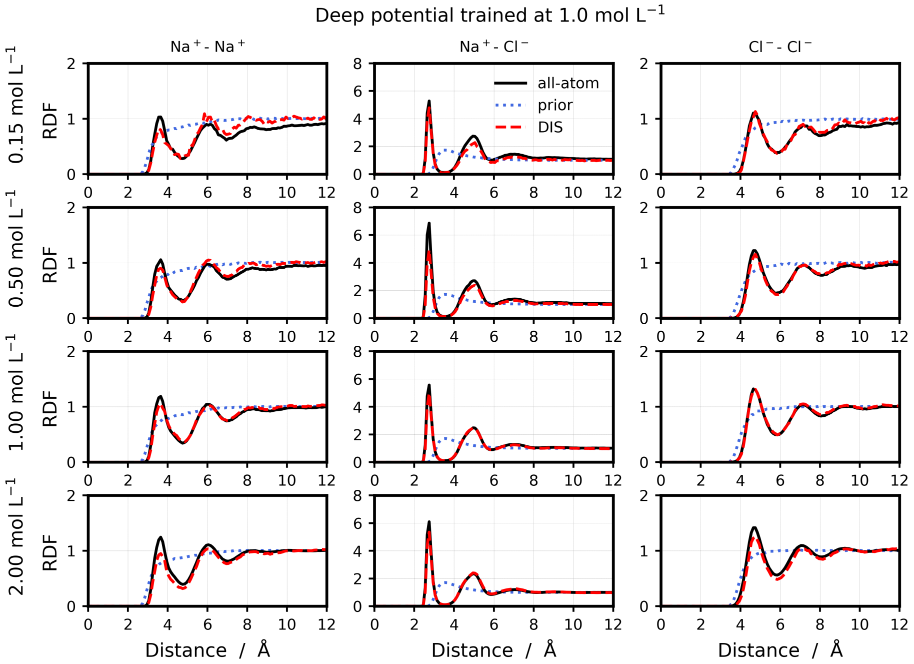

Figure S4: Radial Distribution Functions (RDFs) for the Na^+–Na^+, Na^+–Cl^- and Cl^-–Cl^- pairs and concentrations 0.15, 0.5, 1.0 and 2.0 mol L^{-1}. The deep potential has been trained at 1.0 mol L^{-1}. The all-atom, prior, and DIS models are shown in black, blue, and red, respectively.

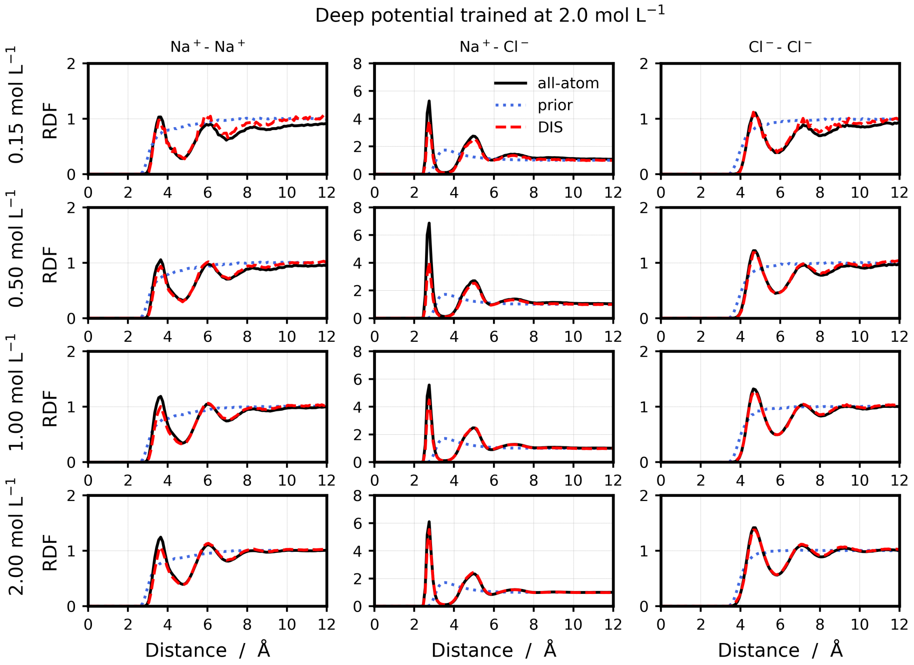

Figure S5: Radial Distribution Functions (RDFs) for the Na^{+}–Na^{+}, Na^{+}–Cl^{-} and Cl^{-}–Cl^{-} pairs and concentrations 0.15, 0.5, 1.0 and 2.0 mol L^{-1}. The deep potential has been trained at 2.0 mol L^{-1}. The all-atom, prior, and DIS models are shown in black, blue, and red, respectively.

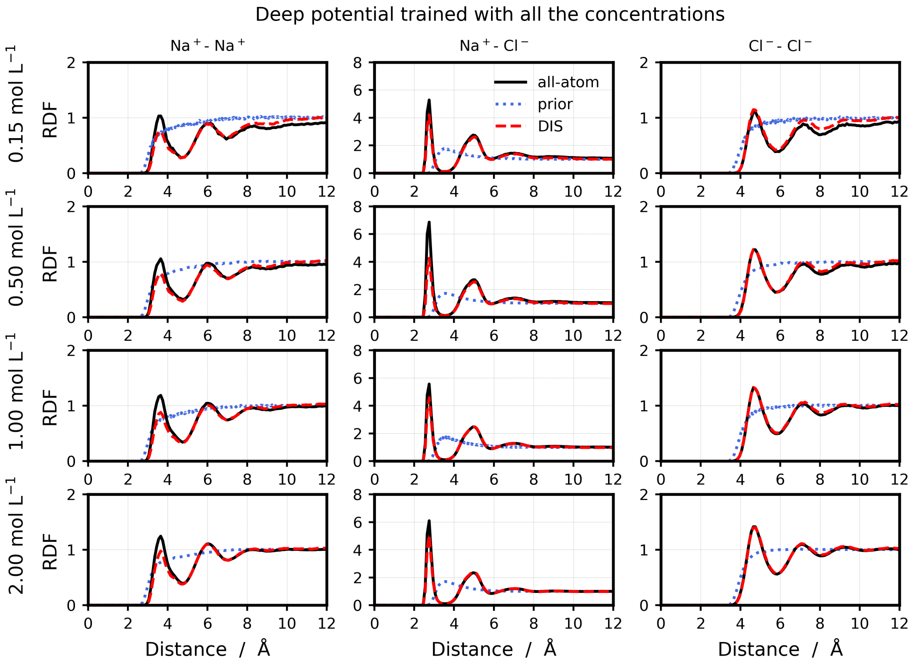

Figure S6: Radial Distribution Functions (RDFs) for the Na^{+}–Na^{+}, Na^{+}–Cl^{-} and Cl^{-}–Cl^{-} pairs and concentrations 0.15, 0.5, 1.0 and 2.0 mol L^{-1}. The deep potential has been trained with all the concentration configurations. The all-atom, prior, and DIS models are shown in black, blue, and red, respectively.

4.2 DNA molecule in an aqueous NaCl salt solutions of 0.5 mol L^{-1}

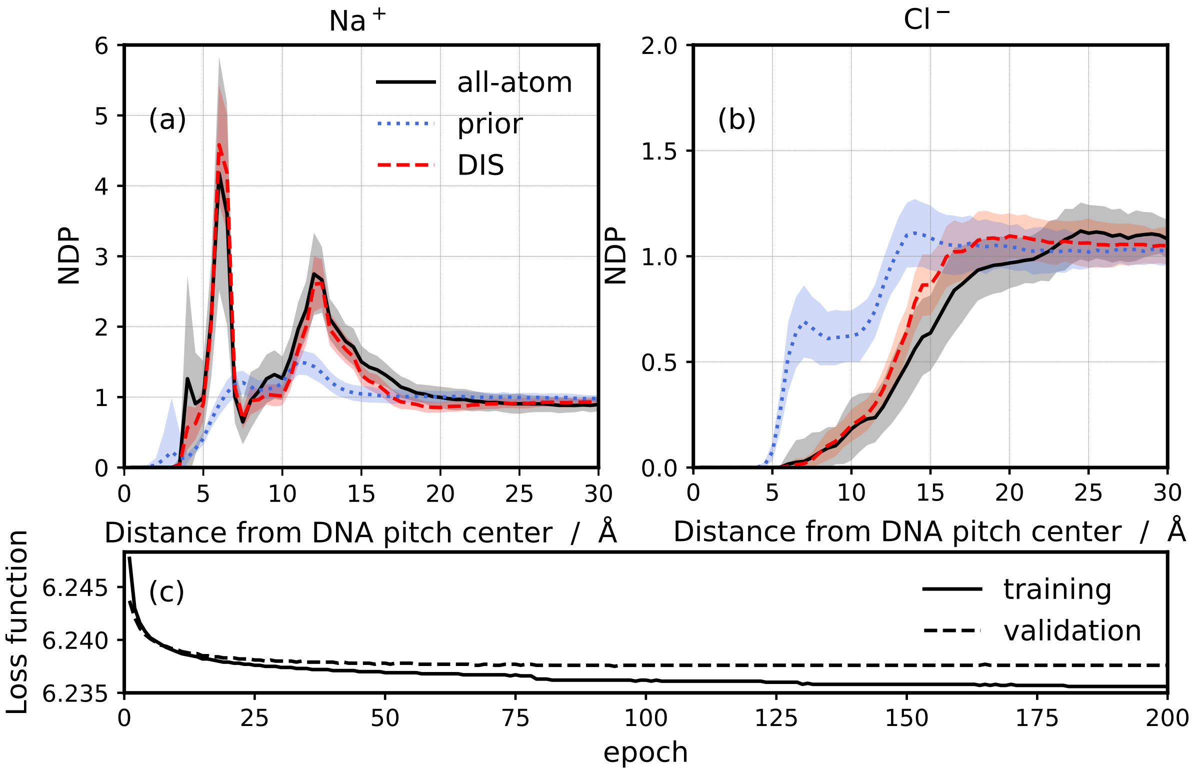

Figure S7: Cylindrical Normalized Density Profiles (NDP) for Na^+ (a) and Cl^- (b) from the center-of-mass of the DNA molecule. The results are shown for the all-atom (black), prior (blue), and DIS (red) simulations at 0.5 mol L^{-1}. The colored areas represent the standard deviation with block averaging of 1 ns. The training (solid) and validation (dashed) loss function as a function of the epochs (c).

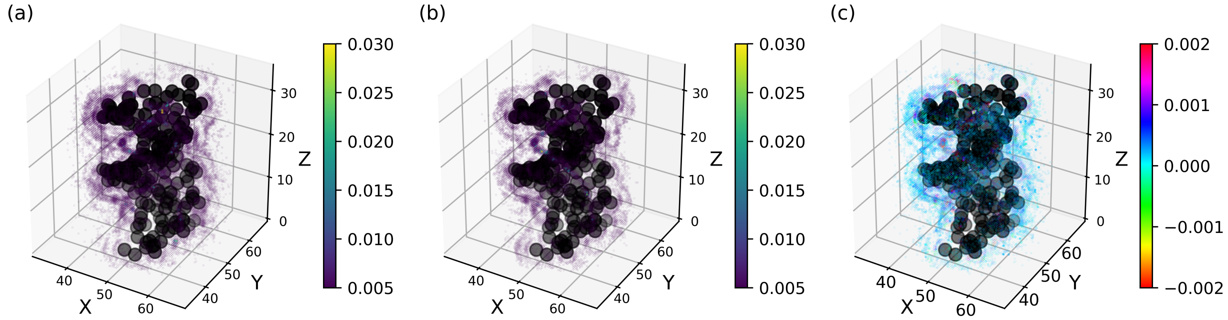

Figure S8: 3D distribution of Na^+ cation around the DNA pitch in a NaCl aqueous solution at 0.5 mol L^{-1} for the all-atom (a), DIS (b), and the difference between both simulations (c). The distribution is normalized over the trajectory and the total number of cations. For clarity, only the positions with a sufficient probability are represented. The black beads represent the coarse-grained resolution of the DNA molecule.

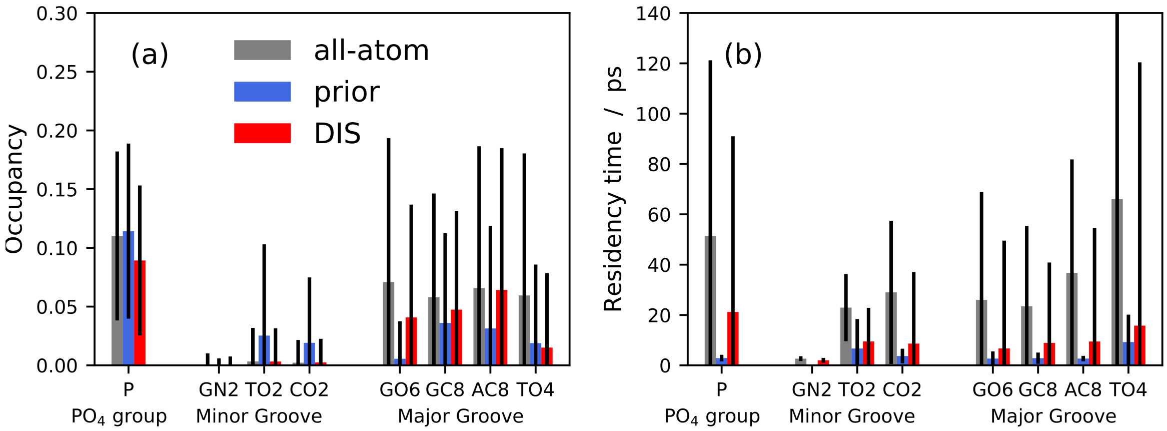

Figure S9: Average occupancy (a) and residence times (b) of Na^+ ions in the first hydration shell of the atoms of DNA at 0.5 mol L^{-1}. The error bars denote the standard deviation. The fast fluctuations (< 1 ps) are omitted in the residence time calculation.

4.3 Aqueous NaCl salt solutions at 5 mol kg^{-1}

To emphasize the need for an accurate description of the n-body term of the potential of mean force, we performed a comparison between our DIS model and two other implicit models. The first one is a transferable effective potential calculated at infinite dilution and using correction terms.[3] The second potential is calculated by a direct IBI potential calculated from RDFs obtained at 5.0 mol L^{-1} by using the STOCK software.[4]

4.3.1 all-atom molecular dynamics with an explicit water model

The simulations have been carried out using LAMMPS.[5] Newton's equations of motion are integrated using the Velocity Verlet integrator[6,7] with a 1 fs timestep. Simulations are performed at a temperature of 298 K. For all-atom simulations, we employ Nosé-Hoover thermostat[8] with a coupling constant of 0.1 ps. In the case of the NPT simulations maintained at 1.0 bars, we additionally use the Nosé-Hoover barostat with coupling constant 1.0 ps. The non-bonded interactions are calculated within cutoff distances of 0.9 and 1.2 nm respectively for the LJ and the electrostatic potential. The electrostatic interactions beyond the cutoff are corrected with the PPPM solver .[9] The force field and the box composition followed the details of the paper.[3] The water molecules are described by the SPC/E water model[10] and NaCl ions are described by the parameter of.[11] An equilibration in NPT of 5 ns was performed followed by a run in NVT of 12 ns. The forces on ions and the position were saved every 0.2 ps.

4.3.2 Direct Iterative Boltzmann Inversion potential

The effective potential between ions was calculated from the all-atom MD with an explicit water model. we use the Software STOCK[4] in order to perform the iterative Boltzmann inversion method[12] and obtain the effective potential U:

$$U_{i+1} = U_i + k_{\mathrm{B}}T \left[\frac{\rho_i(r)}{\rho_{\mathrm{target}}(r)} \right] \tag{S5}$$

tool provides a simple and straightforward way to calculate effective potentials for coarse-grained simulations using the iterative Boltzmann inversion method. For the simulation, we use the Langevin thermostat with a coupling constant 0.1 ps.[13] The simulations are performed under periodic boundary conditions. The cubic box edge is 5.7 nm. To analyze the properties of the trained DIS model, we perform 5 ns NVT simulations after a 1 ns equilibration.

4.3.3 Transferable effective pairwise potential

The effective ion-ion potentials[3] were calculated at infinite dilution using the method defined by Hess *et al.*[14] The effective potentials are computed by constraining the distance between the two ions thanks to the linear constraint solver (LINCS) algorithm.[15] The free energy difference is then obtained by integrating the average constraint force f_c

$$U\left(r_2\right) - U\left(r_1\right) = \int_{r_1}^{r_2} \left\langle f_c \right\rangle_r \, \mathrm{d}r \tag{S6}$$

At long-range, the effective potential is close to a Coulomb interaction, typically between 10 and Å. The total effective potential U_p can be express as

$$U_p(r) = \begin{cases} \int_{r_m}^{r} \left[\left\langle f_c \right\rangle_s + \frac{2k_\mathrm{B}T}{s}\right] \mathrm{d}s + \frac{q_1 q_2}{4\pi\varepsilon_0\varepsilon_r r_m} & r < r_m \\ \frac{q_1 q_2}{4\pi\varepsilon_0\varepsilon_r r} & r \geq r_m \end{cases} \tag{S7}$$

where T is the temperature, k_B the Boltzmann constant, $\frac{2k_\mathrm{B}T}{s}$ is the entropic correction, ε_0 and ε_r are the dielectric permittivity of the vacuum and of the SPC/E water model[16] and r_m is a cutoff-distance equal to 12 Å. The RDFs were extracted from the implicit simulation using a so-called "RDF-refined" correction (Figure 9[3]). By definition, this potential is strictly pairwise.

4.3.4 DIS potential

Hyperparameters for MLP 60K structures have been randomly split into training (80%) and validation (20%) datasets. The cutoff distance was set to 9.0 Å. We set the trainable Bessel functions to 8 with a cutoff polynomial envelope function using $p = 12$. One layer with 8 features with an even parity to be sufficient. The parameter $l_{\max}$ is 2, increasing the value did not improve the fit. A 2-body latent multi-layer perceptron consists of 2 hidden layers of dimensions [32,64] with a SILU non-linearity that is used for all the concentrations. The hidden layer dimension of the latent multi-layer perceptron is tripled. The final edge energy multi-layer perceptron has one hidden layer of 32. The same Allegro architecture can be used for all concentrations, simplifying the hyperparameter studies. The learning rate is set to 0.002 and reduced using an on-plateau scheduler based on the validation loss with a patience of 20 and a decay factor of 0.75. The batch size is 10.

Computational details For simulations employing the DIS model, we use the Langevin thermostat with a coupling constant 0.1 ps.[13] The Lennard-Jones parameters are reported in table S1. The dielectric value is 73 corresponding to the calculated value of SPC/E water model.[16] Due to the very high concentration, the electrostatic interactions beyond the cutoff are corrected with the PPPM solver .[9] The simulations are performed under periodic boundary conditions. The cubic box edge is 5.7 nm. To analyze the properties of the trained DIS model, we perform 10 ns NVT simulations after a 1 ns equilibration.

4.3.5 Structural properties results

The structural properties, *i.e.* the RDFs, are calculated from the explicit and implicit solvent simulations and plotted in Figure S10 For the transferable potential (green), the Na^+–Cl^- is well reproduced. For Na^+–Na^+, the peak at 4.38 Å is not reproduced, however, the intensity of the RDF is close to the all-atom result. For Cl^-–Cl^- interactions, peak at 4.40 Å is not reproduced and the intensity of the peak at 5.2 Å is a little bit higher. The RDFs show

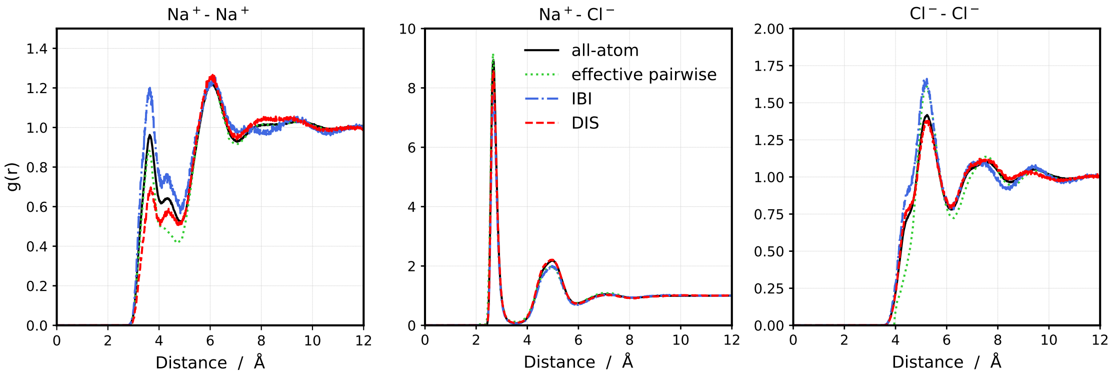

Figure S10: Radial Distribution Functions (RDFs) for the Na^+–Na^+, Na^+–Cl^- and Cl^-–Cl^- pairs at 5.0 mol kg^{-1}. The results are shown for the all-atom (black), the effective pairwise (green), IBI (blue), and DIS (red) models. The effective pairwise results have been taken from the publication[3] (Figure 9)

that some configurations for both ions are not well described. For the IBI potential, the structural properties are reproduced with good accuracy for Na^+–Cl^- and Cl^-–Cl^- but it appears for Na^+–Na^+ that some configurations are well described as they could be. Also, the electrostatic interaction is not explicitly calculated which could explain the difference in intensity at long distances for Na^+–Na^+ and Cl^-–Cl^-. For the DIS model, Na^+–Cl^- and Cl^-–Cl^- are in very good agreement with the all-atom results. For Na^+–Na^+ interaction, despite a lower intensity for the peaks at 3.66 and 4.38 Å, which are also not correctly fit by the other models, the structural properties are in good agreement.

References

(1) Kovaleva, N. A.; Kikot, I. P. K.; Mazo, M. A.; Zubova, E. A. The "sugar" coarse-grained DNA model. *J. Mol. Model.* **2014**, *23*, 1–16.

(2) Musaelian, A.; Batzner, S.; Johansson, A.; Sun, L.; Owen, C. J.; Kornbluth, M.; Kozinsky, B. Learning local equivariant representations for large-scale atomistic dynamics. *Nat. Commun.* **2023**, *14*, 579.

(3) Shen, J.-W.; Li, C.; van der Vegt, N. F.; Peter, C. Transferability of Coarse Grained

Potentials: Implicit Solvent Models for Hydrated Ions. *J. Chem. Theory Comput.* **2011**, *7*, 1916–1927.

(4) Bevc, S.; Junghans, C.; Praprotnik, M. STOCK: Structure mapper and online coarse-graining kit for molecular simulations. *J. Comput. Chem.* **2015**, *36*, 467–477.

(5) Thompson, A. P.; Aktulga, H. M.; Berger, R.; Bolintineanu, D. S.; Brown, W. M.; Crozier, P. S.; in 't Veld, P. J.; Kohlmeyer, A.; Moore, S. G.; Nguyen, T. D.; Shan, R.; Stevens, M. J.; Tranchida, J.; Trott, C.; Plimpton, S. J. LAMMPS - a flexible simulation tool for particle-based materials modeling at the atomic, meso, and continuum scales. *Comput. Phys. Commun.* **2022**, *271*, 108171.

(6) Verlet, L.; Levesque, D. On the Theory of Classical Fluids II. *Physica* **1962**, *28*, 1124–1142.

(7) Verlet, L. Computer "Experiments" on Classical Fluids. I. Thermodynamical Properties of Lennard-Jones Molecules. *Phys. Rev.* **1967**, *159*, 98–103.

(8) Evans, D. J.; Holian, B. L. The Nose–Hoover thermostat. *J. Phys. Chem.* **1985**, *83*, 4069–4074.

(9) Eastwood, J.; Hockney, R.; Lawrence, D. P3M3DP—The three-dimensional periodic particle-particle/ particle-mesh program. *Comput. Phys. Commun.* **1980**, *19*, 215–261.

(10) Zielkiewicz, J. Structural properties of water: Comparison of the SPC, SPCE, TIP4P, and TIP5P models of water. *J. Chem. Phys.* **2005**, *123*, 104501.

(11) Weerasinghe, S.; Smith, P. E. A Kirkwood–Buff derived force field for sodium chloride in water. *J. Chem. Phys.* **2003**, *119*, 11342–11349.

(12) Reith, D.; Pütz, M.; Müller-Plathe, F. Deriving effective mesoscale potentials from atomistic simulations. *J. Comput. Chem.* **2003**, *24*, 1624–1636.

(13) Schneider, T.; Stoll, E. Molecular-dynamics study of a three-dimensional one-component model for distortive phase transitions. *Phys. Rev. B* **1978**, *17*, 1302 – 1322.

(14) Hess, B.; Holm, C.; van der Vegt, N. Osmotic coefficients of atomistic NaCl (aq) force fields. *J. Chem. Phys.* **2006**, *124*, 164509.

(15) Hess, B.; Bekker, H.; Berendsen, H. J. C.; Fraaije, J. G. E. M. LINCS: A linear constraint solver for molecular simulations. *J. Comput. Chem.* **1997**, *18*, 1463–1472.

(16) Kadaoluwa Pathirannahalage, S. P.; Meftahi, N.; Elbourne, A.; Weiss, A. C. G.; McConville, C. F.; Padua, A.; Winkler, D. A.; Costa Gomes, M.; Greaves, T. L.; Le, T. C.; Besford, Q. A.; Christofferson, A. J. Systematic Comparison of the Structural and Dynamic Properties of Commonly Used Water Models for Molecular Dynamics Simulations. *J. Chem. Inf. Model.* **2021**, *61*, 4521–4536.
